# Structural and molecular basis of interaction of HCV non-structural protein 5A with human casein kinase 1α and PKR

**DOI:** 10.1186/1472-6807-12-28

**Published:** 2012-11-13

**Authors:** Govindarajan Sudha, Subburaj Yamunadevi, Nidhi Tyagi, Saumitra Das, Narayanaswamy Srinivasan

**Affiliations:** 1 Molecular Biophysics Unit, Indian Institute of Science, Bangalore, 560 012, India; 2 German cancer research center, Bioquant, Heidelberg, Germany; 3 European Bioinformatics Institute, Hinxton, Cambridge, UK; 4 Department of Microbiology and Cell Biology, Indian Institute of Science, Bangalore, 560 012, India

**Keywords:** Casein kinase 1α, Hepatitis C virus, Interferon therapy, Kinase-substrate complex, Non-structural protein 5A, Protein kinase R

## Abstract

**Background:**

Interaction of non-structural protein 5A (NS5A) of Hepatitis C virus (HCV) with human kinases namely, casein kinase 1α (ck1α) and protein kinase R (PKR) have different functional implications such as regulation of viral replication and evasion of interferon induced immune response respectively. Understanding the structural and molecular basis of interactions of the viral protein with two different human kinases can be useful in developing strategies for treatment against HCV.

**Results:**

Serine 232 of NS5A is known to be phosphorylated by human ck1α. A structural model of NS5A peptide containing phosphoacceptor residue Serine 232 bound to ck1α has been generated using the known 3-D structures of kinase-peptide complexes. The substrate interacting residues in ck1α has been identified from the model and these are found to be conserved well in the ck1 family. ck1α – substrate peptide complex has also been used to understand the structural basis of association between ck1α and its other viral stress induced substrate, tumour suppressor p53 transactivation domain which has a crystal structure available.

Interaction of NS5A with another human kinase PKR is primarily genotype specific. NS5A from genotype 1b has been shown to interact and inhibit PKR whereas NS5A from genotype 2a/3a are unable to bind and inhibit PKR efficiently. This is one of the main reasons for the varied response to interferon therapy in HCV patients across different genotypes. Using PKR crystal structure, sequence alignment and evolutionary trace analysis some of the critical residues responsible for the interaction of NS5A 1b with PKR have been identified.

**Conclusions:**

The substrate interacting residues in ck1α have been identified using the structural model of kinase - substrate peptide. The PKR interacting NS5A 1b residues have also been predicted using PKR crystal structure, NS5A sequence analysis along with known experimental results. Functional significance and nature of interaction of interferon sensitivity determining region and variable region 3 of NS5A in different genotypes with PKR which was experimentally shown are also supported by the findings of evolutionary trace analysis. Designing inhibitors to prevent this interaction could enable the HCV genotype 1 infected patients respond well to interferon therapy.

## Background

Hepatitis C virus (HCV) which belongs to the family *flaviviridae,* causes chronic liver disease, liver cirrhosis and hepatocellular carcinoma in humans [1]. Non-structural protein 5A (NS5A) of HCV is involved in regulating HCV RNA replication [2] and evading the interferon induced antiviral response [3] by interacting with human protein kinases as the viral genome does not encode any kinase [4].

Structural information of NS5A at atomic level is known only for the N terminal part of the protein. Residues 5–25 of NS5A adopts α-helical conformation which is amphipathic and is embedded in a phospholipid bilayer on one side and the other side has conserved polar residues [5]. Residues 36–198 of NS5A adopt a novel zinc coordination motif which forms a homodimer and is organized into different forms to perform different roles such as viral replication and viral assembly [6,7]. Both the regions of NS5A have been shown to be critical for viral replication [5,6]. Experimental work have shown that for the rest of the protein whose structure is currently unknown it is natively unfolded [8], lacks secondary structural elements, is less hydrophobic, has high content of positively charged residues, has low complexity regions and many phosphorylation sites which predominantly occurs in intrinsically disordered regions of the protein [9-11]. Due to these attributes, NS5A which is natively unfolded, attains a stable structural form when it is bound with a protein. The natively unfolded nature of NS5A makes it capable of interacting with many human as well other non-structural proteins of HCV thereby carrying out multiple functions [12].

NS5A interacts with different human kinases namely, casein kinase 1α (ck1α) [13] and Protein kinase R (PKR) [14]. In the present study, the critical kinase and NS5A residues involved in interaction have been predicted by protein structure modeling and sequence analysis.

The Casein kinase 1α – NS5A interaction forms a transient enzyme-substrate complex [13]. Several amino acids in NS5A get phosphorylated by different human kinases [2]. Varying levels of NS5A phosphorylation modulates its interaction with host and other viral proteins during viral replication [2]. Phosphorylation of NS5A serves as a regulatory switch between viral replication and RNA translation and / or packaging [2]. NS5A phosphorylation is observed among the *flaviviridae* family which points out its functional significance for the viral life cycle [15].

Apart from the viral protein NS5A serving as a substrate to CK1α, one of the other substrate for CK1α is tumour suppressor p53 transactivation domain [16]. DNA virus induced stress to human cells leads to phosphorylation of serine 20 of p53 transactivation domain by CK1α. This phosphorylation enables the binding with a co-activator (p300) and stimulates tumour suppressor p53 function [16].

Casein kinase 1 is a serine / threonine protein kinase which is ubiquitously expressed in all the tissues and cellular compartments of eukaryotic organisms. Human casein kinase 1 phosphorylates substrates which are involved in the control of cell differentiation, proliferation, chromosome segregation and circadian rhythm [17]. The catalytic domain of casein kinase 1 is about 300 amino acids long. The catalytic domain of casein kinase 1 is made up of 2 lobes with a cleft between them for the substrate peptide to bind [18].

Human ck1 shows high sequence identity to the other members of casein kinase 1 family [17]. The crystal structure of casein kinase 1, from *Schizosaccharomyces pombe,* bound to ATP [18] closely resembles both cAPK (cAMP dependent kinase) [19] and Cdk2 (cyclin dependent kinase) [20], whose crystal structures have been solved along with substrate peptide and ATP.

Even though NS5A is phosphorylated at several residues by many human kinases [2], clear one-to-one mapping between phosphosite in NS5A and its corresponding human kinase is not completely known. However the human CK1α phosphorylating sites have been mapped to Ser 232 of NS5A [13] and, therefore, phosphorylation by CK1α is the focus of the current study. In the present work, structural model of human CK1α bound to NS5A peptide of HCV containing the phophoacceptor serine 232 has been built using available crystal structures of other kinase-substrate/pseudosubstrate complexes. The aim of building the model of the complex is to understand the structural basis of the NS5A peptide recognition and identification of NS5A interacting ck1α residues. The analogy of ck1α – substrate recognition and interaction is also shown by considering the example of another substrate of ck1α - tumour suppressor p53 transactivation domain, whose crystal structure is known [21].

NS5A also interacts with another protein kinase PKR during the life cycle [14]. NS5A does not get phosphorylated by PKR but only forms a protein-protein complex [14].

Interferons lead to expression of IFN effector proteins like the double-stranded RNA-activated protein kinase (PKR). During viral infection, PKR becomes activated upon viral dsRNA binding, dimerisation and trans-autophosphorylation. PKR in its activated state phosphorylates the eukaryotic translation initiation factor 2 (eIF-2α), thereby inhibiting cellular protein synthesis and blocking viral replication [22].

In the recent past, triple therapy, which includes pegylated interferon along with ribavirin and a HCV protease inhibitor, has shown promising results with high rates of sustained virological response compared to other treatments currently available [23-25]. Combined pegylated interferon, ribavirin therapy provides ~45-50% sustained virological response in genotype-1 infected patients [26-28] while when interferon alone is administered only ~30% of genotype 1 infected patients seem to respond to the therapy [1]. This is mainly because a region in NS5A [interferon sensitivity determining region-ISDR (237–302)] interacts with PKR [dimerisation domain (244–296)], thereby preventing PKR dimer formation and activation [14]. Additionally, another region in NS5A, referred as the Variable-region 3 (V3) (381–407) is located downstream to ISDR which is also involved in the interaction and inhibition of PKR [29]. Interestingly, more than 60% of genotype 2 and 3 patients respond well to the interferon therapy. One of the reasons for this is that NS5A 2a/3a are unable to bind and inhibit PKR efficiently [30].

An attempt has been made to narrow down the region in NS5A and predict critical residues in NS5A 1b responsible for interaction with PKR by two independent approaches. Firstly, due to putative shared binding region of NS5A 1b and PKR protomer with another PKR protomer [14], one can hypothesize that structural features of PKR dimer interface and PKR interacting region in NS5A 1b would be similar. The unstructured region of NS5A 1b [9-11] could attain a stable structural form and have similar binding surface like that of PKR protomer as it binds to the other PKR protomer. Some of the critical PKR interacting residues of NS5A has been identified using this approach.

Further, as the binding of NS5A with PKR is primarily genotype specific [14,29,30], evolutionary trace analysis [31,32] has been used to identify amino acid residues in NS5A specific for genotype 1 that interacts with PKR and residues in NS5A specific to genotype 2A and 3A which is potentially incapable of binding and inhibiting PKR efficiently.

Details of the NS5A and human kinase interacting residues shown in this study can provide insights to design antivirals to inhibit these interactions.

## Results and discussion

### Homology modeling of human casein kinase 1α

**Figure 1:**
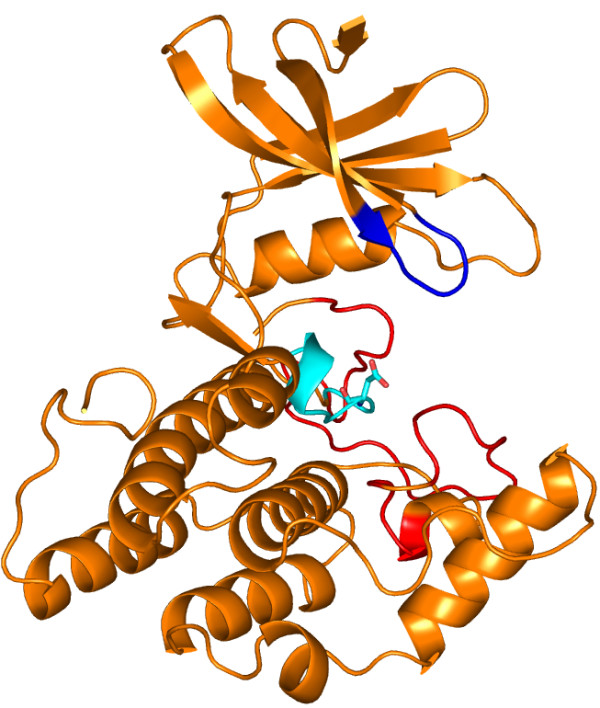
**Homology model of CK1α.** Catalytic base Asp 136 shown in stick representation in the catalytic loop is coloured in cyan. Activation loop and glycine loop in the kinase are coloured red and blue respectively. All the figures of protein structures in this analysis were generated using PyMOL [33].

Casein kinase 1 from *Schizosaccharomyces pombe,* for which a crystal structure is available in complex with MgATP at 2Å resolution (PDB id:1CSN) [18], showed 45.5% sequence identity with the sequence of human ck1α. Casein kinase 1 from *Schizosaccharomyces pombe* can be considered as a best template, as the kinase is in the active conformation so that the substrate is accessible to get phosphorylated by the kinase. The homology model of the kinase domain of ck1α is shown in Figure 1.

An insertion region (refer Methods section) away from critical sites of kinase and the substrate interacting region has not been modeled due to potentially poor reliability of the modeled conformation. These inserts are unlikely to have any influence on the substrate binding.

The sequence of human ck1α can be accommodated comfortably in the bilobal structure which is common to all Ser/Thr/Tyr protein kinases [34]. It is ensured that all the expected features such as presence of nucleotide binding site in the smaller lobe and the characteristic glycine-rich ATP binding loop (GSGSFG) are present in the model at the correct positions in the 3-D structure.

### Modelling of NS5A peptide bound to human casein kinase 1α model

Protein kinases recognize their substrates usually by means of a substrate recognition motif surrounding the phosphoacceptor residue [35]. Substrates of Casein kinase 1 have Asp/Glu or a phosphorylated amino acid residue located three residues upstream of phosphoacceptor serine and this residue is termed as the substrate constraint residue [13,35]. ck1α phosphorylates NS5A which contains the substrate constraint residue phosphoserine at the position 229. This residue is three residues upstream to Ser 232 targeted for phosphorylation by ck1α thus defining a substrate recognition motif of -S(P)-X-X-S, where X refers to any amino acid and S(P) refers to phosphoserine [13]. The substrate sequence motif for cAMP dependent kinase (cAMP) is R-R/K-X-S where S at the fourth position is the target site for phosphorylation [19]. It must be noted that both in CK1α and cAMP kinase, substrate specificity sequence pattern constraints are imposed in first and fourth positions of the motif. Given the conserved nature of catalytic and ATP anchoring residues in the 3-D structures of cAMP and CK1α, it is expected that phospho acceptor site and the residue two positions upstream to the phosphor-acceptor site are oriented in the similar way with respect to their respective kinases. This leaves little scope for the variation in the backbone conformation between the first and fourth residue positions of the two substrates. Therefore, NS5A peptide S(P)SAS has been modeled on to the structural model of ck1α which was generated based on the crystal structure of cAMP dependent kinase – peptide inhibitor (PKI) complex (PDB id: 1ATP) [19]. (Refer Methods section for details). Hence the conformation of NS5A peptide is based on the conformation of peptide inhibitor of cAMP dependent kinase.

**Figure 2:**
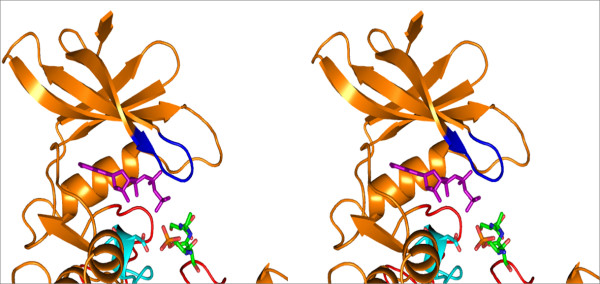
**Model of human CK1α bound to NS5A peptide whose phosphoserine is topologically equivalent to arginine in substrate peptide bound to cAMP dependent kinase.** Stereo figure showing modelled position of the side chain of phosphoserine 229 of NS5A in the same orientation as its topologically equivalent residue (an Arg) of the pseudosubstrate of cAPK (cAMP dependent kinase). This brings the side chain of phosphoserine 229 coloured in green unfavourably proximal to a like charged side chain of Asp 140 coloured in cyan. Therefore, the side chain of phosphoserine is unlikely to be oriented in the same way as the topologically equivalent Arg in the pseudosubstrate of cAMP dependent kinase. After the remodelling of the side chain of phosphoserine 229 it points towards the putative substrate interacting residues Arg 214, Lys 260, Gly 251 and makes favourable ionic interaction as shown in Figure 3.

The modeled complex of NS5A peptide bound to ck1α revealed that the phosphoserine is close to Asp 140 (Figure 2), which is unfavourable due to the proximity of two strongly negative charges. Multiple sequence alignment (MSA) among the casein kinase 1 family also revealed that the Asp is conservatively substituted to Glu as shown in the Additional file 1. Therefore it was inferred that the conformation of side chain of phosphoserine is unlikely to be similar to that of Arg in "equivalent position" of cAMP dependent kinase [19]. The conformation of side chain of phosphoserine was modelled in such a way that the negatively charged phosphoserine can make favourable ionic interactions with positively charged residues in the vicinity.

**Figure 3:**
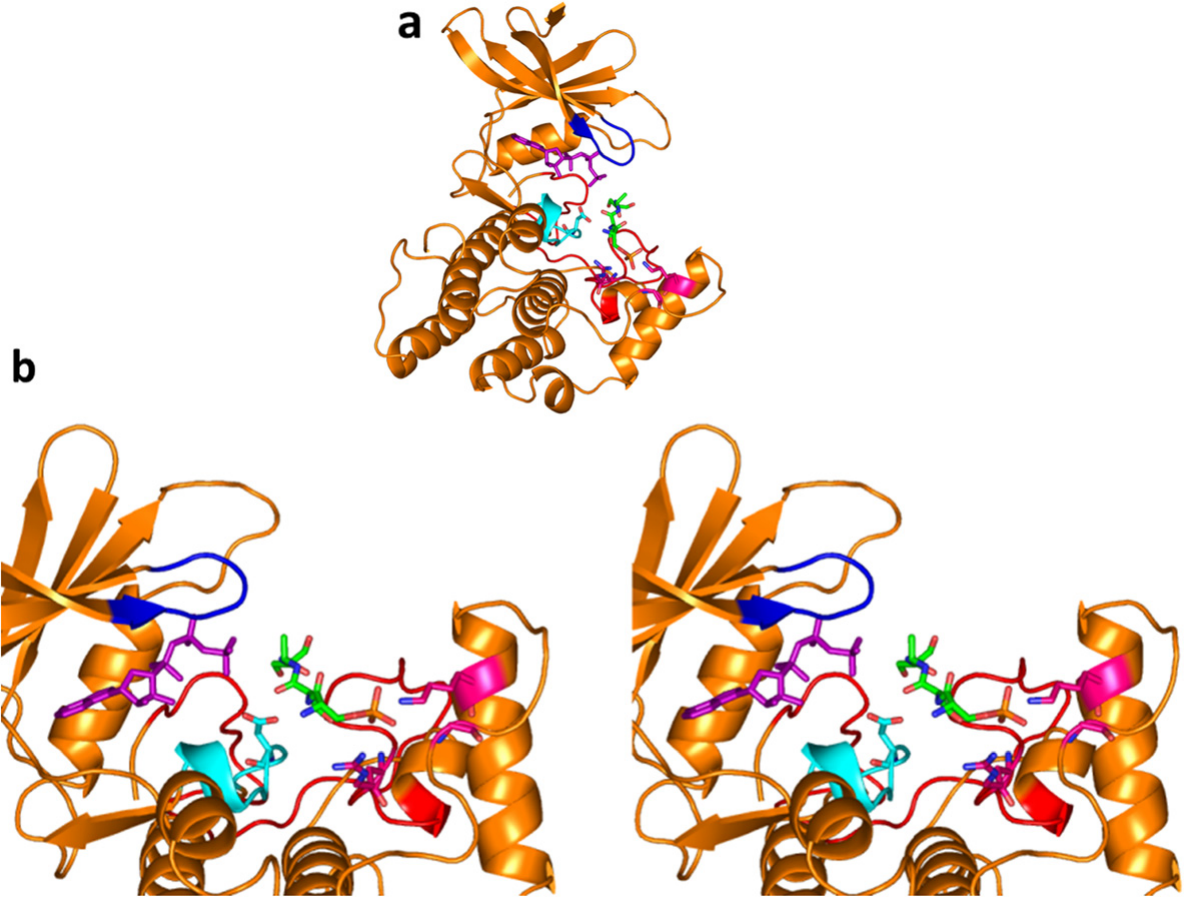
**Model of human CK1α with NS5A substrate peptide (green) and ATP molecule (purple). a**: Catalytic base Asp 136 in catalytic loop (cyan), NS5A peptide (green) containing the phosphoacceptor serine 232 and phosphoserine 229 are shown in stick representation. Glycine loop (blue) is also shown. **b**: Stereo figure showing the zoomed in view of the substrate interacting kinase residues Arg 214, Lys 260, Gly 251 (magenta) making ionic interactions with phosphoserine of NS5A peptide. Ser 230 of NS5A peptide interacts with Thr 212 by means of hydrogen bonding. Phospho acceptor serine 232 of NS5A is proximal to the γ phosphate of ATP molecule and the catalytic base Asp 136 to carry out phosphorylation.

From the modeled complex structure, the positively charged residues in close vicinity to phosphoserine were found to be side chains of Arginine 214, Lysine 260 and main chain nitrogen of Glycine 251 which are capable of making ionic interactions as shown in Figure 3. These residues are critical for the proper positioning of the HCV NS5A for the phosphorylation to occur at Ser 232.

**Figure 4:**
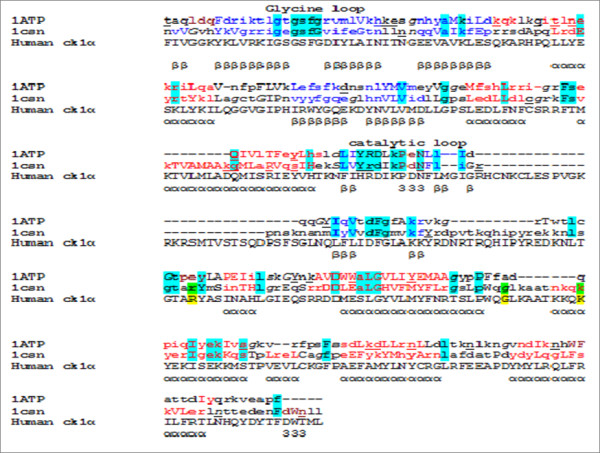
**Protein sequence and structure alignments.** Sequence alignment between template sequence (yeast casein kinase 1) and CK1α shows 45.5% sequence identity. Protein structure alignment using DALI [36] between cAMP dependent kinase (1ATP) [19] and yeast casein kinase (1CSN) [18] shows that the critical residues which are highlighted in blue are topologically equivalent. The substrate interacting residues in ck1α is highlighted in yellow and its structurally equivalent substrate interacting residues as proposed by the crystal structure study of yeast casein kinase 1 is highlighted in green. This figure was produced using JOY [37]. Key to JOY notation: Solvent inaccessible - UPPER CASE (O); Solvent accessible - lower case (o); Positive φ - *italic* (*o*); *Cis*peptide – breve (õ); Hydrogen bond to other side chain, tilde (ö); Hydrogen bond to main chain amide, **bold (o)**;Hydrogen bond to main chain carbonyl - underline (o); Disulphide bond, -cedila (ç).

Multiple sequence alignment was used to check the structural importance of these residues within the casein kinase 1 family, as shown in the Additional file 1. Arginine is completely conserved across the casein kinase 1 family. Lysine is conservatively substituted to arginine across the casein kinase 1 family. The glycine does not show absolute conservation, but this does not affect its interaction with phosphoserine because it is the main chain nitrogen that is involved in the interaction. Previous crystal structure studies of casein kinase 1 from *Schizosaccharomyces pombe* used sulphate anion that can mimic the binding of a phospho amino acid. The authors proposed that arginine 183, lysine 229 and glycine 220 are substrate interacting residues [18]. These residues are found to be topologically equivalent to the residues that had been identified in the model as substrate interacting residues as shown in the Figure 4. Sequence alignment between casein kinase 1 from *Schizosaccharomyces pombe* and human ck1α and structure alignment of casein kinase 1 from *Schizosaccharomyces pombe* [PDB id: 1CSN] [18] and cAMP dependent kinase [PDB id: 1ATP] [19] is shown in the Figure 4.

The conformation of NS5A motif S (P) SAS as shown in the Figure 3, would be functional irrespective of its entire tertiary structure context in the protein. The interactions shown in the modeled complex is likely to be identical when the structure of the entire NS5A is considered. This modeled complex has helped us to find out the substrate interacting residues in kinase with the substrate constraint residue phosphoserine which is common not only for NS5A but many other substrates of ck1α. However, it should be noted that the sequence specificity (substrate recognition motif) of different kinases for phosphorylation is different. Therefore, we believe that the modeled structure of CK1α -NS5A complex presented in our work cannot be assumed for interaction of other kinases with NS5A.

Kinase inhibitors to prevent NS5A phosphorylation could severely affect the biological role of ck1α as it is involved in cell cycle control, apoptosis, Wnt signaling cascade for cell proliferation, differentiation and, deregulation of ck1α expression has been linked to Alzheimer’s and Parkinson’s disease [17].

Other critical interactions specific to only NS5A and ck1α is not understood due to the unavailability of structural details of entire NS5A protein. A small molecule targeted to specifically bind to the suggested part of NS5A to prevent its interaction with ck1α could be very useful.

### Modeling of p53 transactivation domain bound to human casein kinase 1α

The crystal structure of p53 transactivation domain [21] contains the casein kinase 1 substrate consensus motif (ETFS). The substrate consensus motif contains the phospho acceptor Ser 20 and glutamic acid, the substrate constraint residue spaced three residues upstream to Ser 20 [16]. This glutamic acid is equivalent to phosphoserine at position 229 in NS5A.

**Figure 5:**
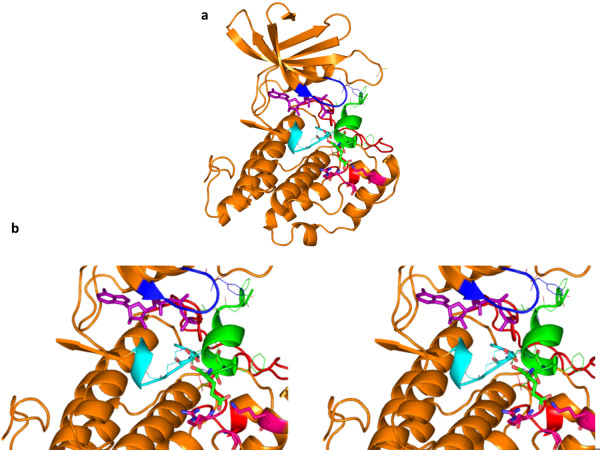
**Model of CK1α (orange) with crystal structure of p53 transactivation domain (green) and ATP molecule (purple). a**: Catalytic base Asp 136 in catalytic loop of kinase is coloured in cyan. p53 transactivation domain coloured in green consists of phosphoacceptor serine and the substrate constraint residue glutamic acid shown in stick representation. Substrate interacting residues arginine 214, lysine 260 and glycine 251 are shown in magenta and glycine loop is coloured in blue. **b:** Stereo figure showing the zoomed in view of the interaction between crystal structure of p53 transactivation domain and ck1α homology model. Other non-covalent interactions made between the kinase and the substrates are shown as discussed in Table 1.

Modeling of p53 transactivation domain [PDB id: 1YCR] bound to ck1α showed the analogy of substrate recognition and interaction with ck1α. The substrate interacting residues arginine 214, glycine 251 and lysine 260 makes ionic interactions with the substrate constraint residue glutamic acid 17 in the crystal structure of the transactivation domain of p53 tumour suppressor [21] as shown in the Figure 5.

This modeled complex shows identical interactions between the substrate interacting residues of kinase and the substrate constraint residues, which is common to all substrate - ck1α interaction. The other unique non-covalent interactions made by the p53 transactivation domain and kinase are listed in the Table 1.

**Table 1:** List of interactions between human casein kinase 1α and substrate p53 tumour suppressor transactivation domain

**Residues in the crystal structure of p53 transactivation domain**	**Substrate interacting residues of human casein kinase 1α**	**Nature of interaction**
E17	R214, K260, G251	Ionic interaction
T18	T212	Hydrogen bonding
F19	K260	Cation-π interaction
D21	K138	Ionic interaction
K24	D136	Ionic interaction
L25	F28	Hydrophobic interaction
P27	A52, P55	Hydrophobic interaction
E28	R135	Ionic interaction

Interactions observed in the modelled complex of p53 tumour suppressor transactivation domain [21] bound to CK1α model as shown in Figure 5.

### Prediction of PKR interacting residues of NS5A

#### Information derived from crystal structure of PKR dimer and pairwise sequence alignment of NS5A and PKR

Crystal structure of PKR dimer [PDB id: 2a19] [22] was used to identify the interface residues listed in Table 2. Given a reasonable sequence similarity between ISDR of NS5A (237–302) [14] and a stretch of interface in PKR dimeric interface (262–327) [22] we raise the possibility of structural mimicry between a local (ISDR) region of NS5A and a local stretch (interface segment) of PKR. This hypothesis is attractive especially because available experimental data suggests that ISDR is potentially preventing dimerization of PKR [14]. However, the gross similarity in the tertiary structures of PKR and NS5A is not implied.

**Table 2:** List of dimer interface residues in PKR and the nature of interactions between them

**PKR (B chain)**	**PKR (C chain)**	**Nature of interaction**
R262	D266	Ionic interaction
D266	R262	Ionic interaction
H286	C326	Hydrogen bond : NE2 of His with O of Cys
I288	Y300, V309	Hydrophobic interaction
D289	Y323	Hydrogen bond: OD2 of asp with OH of Tyr
Y293	Y323	Hydrophobic interaction
D316	H322	Ionic interaction,
V318	V318	Hydrophobic interaction
H322	D316,Y323	Hydrogen bond: ND1 of his with OD1 of Asp
		NE2 of his with OD1 of Asp
		NE2 of his with O of Tyr
		Ionic interaction
Y323	Y293, H322,D289	Hydrophobic interaction
		Hydrogen bond : O of Tyr with NE2 of His
		OH of Tyr with OD2 of Asp
N324	Y323,N324	Hydrogen bond: ND2 of Asn with O of Tyr
		ND2 of Asn with O of Asn
C326	H286	Hydrogen bond: SG of Cys with NE2 of His

The list of non-covalent interactions at the dimeric interface of PKR [22] has been obtained by using PIC (protein interaction calculator) server [38].

NS5A could have independently evolved the binding features of human PKR which is a mechanism commonly observed in viruses to evade the host system [39]. This assumption is convincing to us because the structural constraint imposed by the binding pocket in the PKR protomer would make the natively unfolded region of NS5A [9-11] to flexibly adopt a structural region that could mimic the binding of PKR protomer.

However, the 3-D structure of NS5A is unlikely to be similar to the tertiary structure of PKR and also a previous study [10] showed that region of NS5A of current interest contains many unstructured regions.

**Figure 6:**
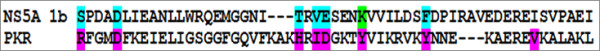
**Pairwise sequence alignment between ISDR (Interferon sensitivity determining region) of NS5A and PKR dimerisation region.** Some of the residues involved in dimerisation of PKR are highlighted in magenta. Equivalent residues in NS5A are highlighted in cyan. Non equivalent residue in NS5A is highlighted in green. The equivalent position for dimer interface residue V is not marked in NS5A sequence because this position is highly variable.

**Figure 7:**
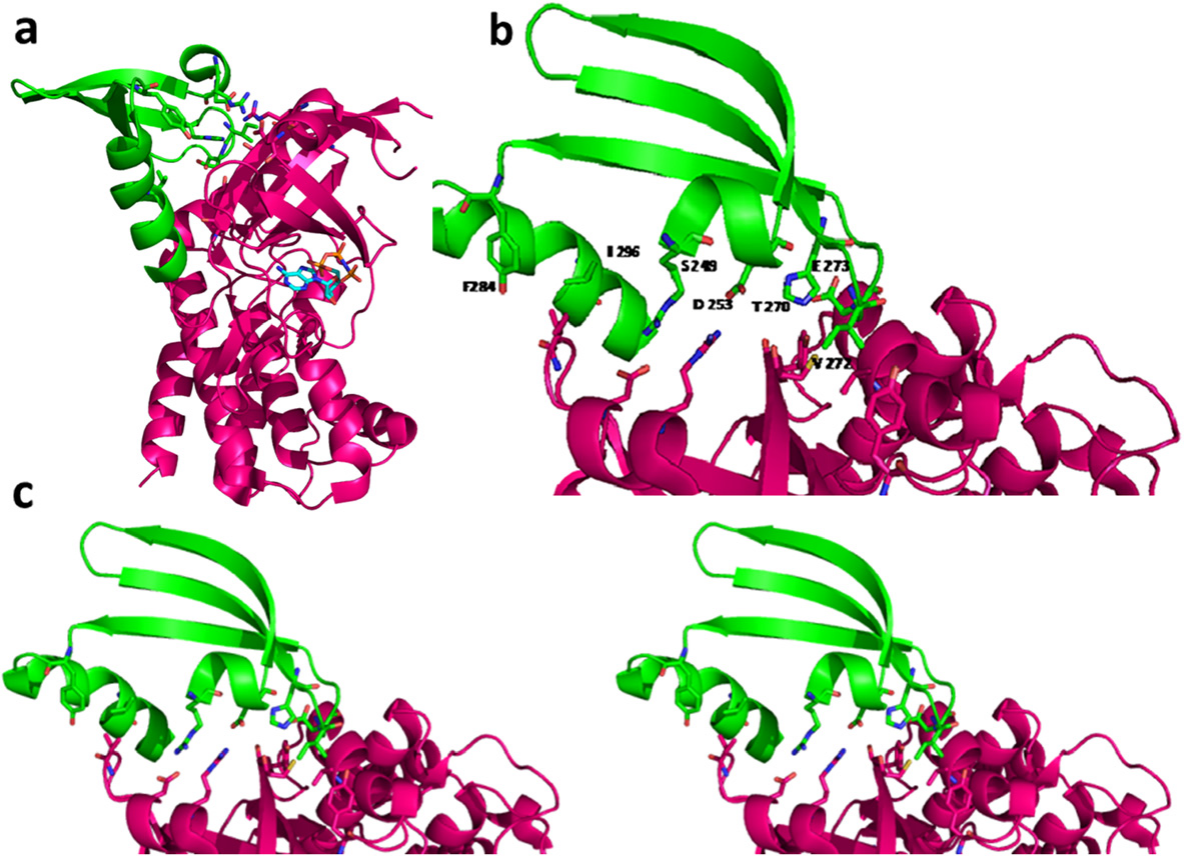
**Binding features of PKR protomer being mimicked by NS5A to interact with another PKR protomer. a**: Binding features of PKR being mimicked by NS5A [14] is coloured in green which is interacting with another PKR protomer coloured in magenta. The dimer interface residues are shown in stick representation. **b**: Zoomed in view with labeled NS5A residues equivalent to the dimer interface residues as per the pairwise sequence alignment shown in Figure 6. **c**: Stereo representation of the image in Figure 7b.

A portion of NS5A ISDR that could mimic the binding of a PKR protomer to other PKR protomer has been shown in the Figure 6 in the form of pairwise alignment. The dimer interface residues of PKR are marked in the alignment and the equivalent residues were checked in NS5A 1b sequence from the alignment. Interestingly, it was found that the equivalent residues in NS5A 1b were mostly biochemically and structurally similar to the dimer interface residues in PKR, as shown in the Figure 7; therefore they may be capable of making identical or similar interactions like PKR protomer. Thus the residues in NS5A 1b equivalent to dimer interface residues of PKR were predicted as PKR interacting residues. They are Ser 249, Asp 253, Thr 270, Val 272, Glu 273, and Phe 284 as shown in the Table 3.

**Table 3:** Dimer interface residues in PKR that are mimicked by the residues in HCV NS5A

**Nature of interaction**	**Dimer interface residues of PKR**	**Equivalent residues in NS5A**
D266 (Ionic interaction)	R262	S249
R262 (Ionic interaction)	D266	D253
C326 (Hydrogen bond : NE2 of His with O of Cys )	H286	T270
Y300, V309 (Hydrophobic interaction)	I288	V272
Y323 (Hydrogen bond: OD2 of Asp with OH of Tyr)	D289	E273
I288, V309 (Hydrophobic interaction)	Y300	F284

The equivalent residues in NS5A to the PKR dimer interface residues are obtained from the pairwise alignment as shown in the Figure 6. The equivalent residues in NS5A which are capable of interacting with PKR mimics the binding of PKR protomer to the other PKR protomer due to the shared binding region of NS5A and PKR protomer with another PKR protomer [14].

The conservation of the predicted PKR interacting residues from all NS5A-1b sequences (genotype 1b −331 sequences) was found to be above 90%. Even though Val 296 is similar to the dimer interface residue in the alignment, it was conserved below 80% and this position is highly variable with substitutions like I, P and M. So this position is not predicted as a PKR interacting residue.

It was further considered that the predicted PKR binding residues from genotype 1b would be different to those equivalent residues in genotype 2a and 3a which are unable to bind and inhibit PKR efficiently. Multiple sequence alignment (Additional file 2) (MSA 1b Vs 3a, 1b Vs 2a) shows different amino acids in genotype 3a and 2a equivalent to the predicted PKR interacting residues in genotype 1b.

### Prediction of PKR interacting residues of NS5A 1b by evolutionary trace analysis

We have also predicted the PKR interacting residues in NS5A of genotype 1b by a computational analysis which is entirely independent of the analysis reported in the previous section. Experiments have shown that PKR interaction with NS5A is primarily genotype specific [14,29,30]. NS5A from genotype 1b is able to efficiently interact and inhibit PKR [14] whereas NS5A from genotype 2a and 3a are unable to interact and inhibit PKR efficiently [29,30].

Evolutionary trace analysis is based on the assumption that residues responsible for functional specificity could undergo many amino acid substitutions, which are responsible for variation in their function [31,32]. The variation in the binding abilities of NS5A across genotypes with PKR can help us to predict the critical residues specific to NS5A 1b that could be involved in interaction with PKR. Further evolutionary trace analysis could enable us to predict NS5A 2A, 3A specific residues which are unable to bind and inhibit PKR efficiently.

The residues unique to ISDR of genotype 1b have been identified when the MSA of genotype 1b was compared to MSA of genotype 3a as shown in the Additional file 2. Analysis of V3 region of NS5A in the two genotypes showed a clear difference. As reported earlier there is an insertion within the V3 region [40] unique to all genotype 3a sequences and also few residues specific to genotype 3a different from genotype 1b. Similar results were observed for genotype 1b versus genotype 2a as shown in the Additional file 2. Long insertion unique to both genotype 2a and 3a within the V3 region and residues unique to both genotype 2a, 3a collectively in ISDR and V3 region differing from genotype 1b have been identified as shown in the Additional file 2.

The list of evolutionary trace residues [Refer Methods section] picked up is provided in Table 4 along with the extent of amino-acid substitution across genotypes shown by means of BLOSUM-62 score. All the trace residues show a high conservation of greater than 90% with very few residues showing greater than 80% conservation in the entire dataset (1b - 337 sequences, 3a −31 sequences, and 2a – 16 sequences).

**Table 4:** Evolutionary trace residues in ISDR and V3 region of NS5A from genotype 1b, 3a and 2a

**Genotype 1b Vs 3a**			**Genotype 1b Vs 2a**			**Genotype 1b Vs 3a and 2a**			
**Trace residues (NS5A -1b)**	**Trace residues (NS5A -3A)**	**Blosum-62 substitution score**	**Trace residues (NS5A -1b)**	**Trace residues (NS5A -2A)**	**BLOSUM-62 substitution score**	**Trace residues (NS5A-1b)**	**Trace residues in (NS5A -3a and 2a)**		**BLOSUM-62 Substitution score**
T244	Q244	0	K240	R240	2	I255	V255	V255	1
H247	R247	0	H247	G247	−2	E256	D256	D256	2
D248	P248	−1	P250	Y250	−3	N276	T276	S272	0,1
S249	H249	−1	A252	V252	−2	E293	D293	D289	2
D253	E253	2	L254	M254	2	-	K384	P380	
I255	V255	1	I255	V255	1	-	P385	P381	
E256	D256	2	E256	D256	2	-	Q386	S382	
G267	S267	0	L260	F260	0	-	E387	G383	
N276	T276	0	W261	-		-	E388	D384	
E293	D293	2	R262	-		A387	G394	G390	0
I302	C302	−1	Q263	-		T292	S399	D395	1,1
G381	P381	−2	E264	-		A293	S400	S396	1
-	K384		I269	V265	1	-	V405	T400	
-	P385		V272	I268	3	S401	-	-	
-	Q386		N276	S272	1	D402	-	-	
-	E387		I280	V276	1	G404	-	-	
-	E388		F284	L280	0	D405	-	-	
A387	G394	0	L287	M283	2				
S390	T397	1	A289	E285	−1				
G391	Q398	−2	D292	S288	0				
T392	S399	1	E293	D289	2				
A393	S400	1	R294	L290	−2				
P396	S403	−1	V298	I294	3				
P397	K404	−1	A300	S296	1				
D398	V405	−3	I302	Y298	−1				
Q399	P406	−1	-	Q378					
D402	P409	−1	-	P379					
D405	E412	2	-	P380					
G407	-		-	P381					
			-	S382					
			-	G383					
			-	D384					
			-	S385					
			-	G386					
			-	L387					
				T389	1				
				A391	1				
				D392	0				
				-					
				E404	2				
				-					
				-					
				-					
				-					
				L407	−4				

Trace residues are picked up if complete/ high conservation (mostly >90% and very few residues showing >80%) or conservative substitution of two different residues or a residue and a gap from both multiple sequence alignment are being compared. The extent of amino acid substitution across genotypes in NS5A has been shown using BLOSUM – 62 substitution score. The trace residues from NS5A genotype1b are predicted to be PKR interacting residues.

It is interesting to note that 4 out of the 6 residues (Ser 249, Asp 253, Val 272, Phe 284) predicted to be PKR interacting residues in NS5A 1b by the method of aligning NS5A 1b and PKR, has also been picked by evolutionary trace analysis. This clearly shows the importance of these residues in binding to PKR.

These observations again emphasizes that both ISDR as well as V3 region are involved in PKR inhibition which are primarily genotype specific as already shown by experimental studies [14,29,30,40] and which is reflected in form of trace residues in this study. The critical residues within ISDR and V3 region involved in inhibition with PKR specific to only NS5A 1b have been identified for the first time by evolutionary trace analysis method. The striking common feature in genotypes 2a and 3a are the presence of the insertion of few residues within the V3 region that have been picked up as trace residues.

The finding from evolutionary trace analysis is consistent with the experiments done previously showing the importance of NS5A ISDR and V3 region to bind to PKR [40].

V3 region in NS5A was found to be crucial for providing specificity to binding/ poor binding efficiency to PKR in the case of genotype 1b and genotype 2a/3a respectively [29,40]. The large number of trace residues been picked up in V3 region as compared to ISDR shows its importance and specificity for PKR binding. Insertion within V3 that is commonly observed in genotype 3a and 2a could make NS5A unable to bind and inhibit PKR efficiently. It was found that ISDR may not be critical for imparting specificity of binding to PKR [40]. Though ISDR from genotype 1b is capable of interacting with PKR, its final interaction is solely dependent on the nature of V3 present [40]. The PKR interacting residues in ISDR picked in this analysis could be involved in stabilizing the protein-protein complex in case of genotype 1b with PKR.

It should be noted that not all the variable positions within NS5A 1b sequences may be completely responsible for their interaction with PKR. Moreover NS5A has binding partners other than PKR [12] and neutral sequence evolution of various serotypes is also possible [41]. Nevertheless, it is attractive to hypothesize that sequence changes primarily in ISDR in NS5A could be responsible for PKR interaction, as previous studies have shown the physical interaction between the ISDR region of NS5A and PKR [14]. Therefore, mutagenesis experiments can be performed based on the predicted PKR interacting NS5A 1b residues to validate and filter them.

## Conclusions

Structural modeling has provided insights about the structural basis of interaction and recognition of NS5A peptide containing the phosphorylation motif with ck1α. This shows the viral mimicry of the substrate recognition motif for human ck1α. Mutation of Arg 214 and Lys 260 which are predicted to interact with phosphorylated Ser in ck1α could serve as an interesting validation of the model.

Analogy of kinase recognition and substrate interaction is shown by considering another substrate of ck1α which is p53 tumour suppressor transactivation domain [16] whose crystal structure is known [21].The residues found to interact in ck1α with the substrate constraint residue are common in the viral protein HCV NS5A and human p53 tumour suppressor transactivation domain.

NS5A phosphorylation by ck1α serves as a regulatory switch between viral replication and RNA translation/packaging [2]. On the other hand, p53 phosphorylation at the transactivation domain by ck1α upon DNA virus induced stress is also shown [16,42]. It is well known that phosphorylated p53 triggers apoptosis in infected cells and serves as a host response during viral infection [43]. Interestingly, previous experiments have shown that NS5A binds and sequesters p53 transactivation domain and thereby prevents apoptosis to counteract the host response [44,45].

It is interesting to speculate that HCV infection could trigger phosphorylation of p53 by ck1α as a host response which gets counteracted by the HCV NS5A sequestering the p53 to prevent apoptosis [44,45].

Thus, identification of residues in human ck1α which interacts with NS5A and p53 in this work could provide further insights to understand the interplay between ck1α and its two substrates (NS5A and p53) which could influence the outcome of the viral infection.

Two different methods have been used to narrow down the range in NS5A which might interact with PKR. Critical residues responsible for interaction of NS5A 1b with PKR and residues unable to bind and inhibit PKR efficiently in NS5A 2a/3a have been predicted for the first time. The functional significance and nature of interaction of ISDR and V3 region in NS5A with PKR which was previously identified experimentally [14,29,30,40] are supported by evolutionary trace analysis.

Even though the clinical response to interferon therapy has been studied for other HCV genotypes like 4, 5 and 6 [46], the molecular interaction with PKR is not known. The findings of the evolutionary trace analysis can be compared to further predict if NS5A in these genotypes would interact with PKR and predict the PKR interacting residues in them.

Recent work on evolution of PKR showed that each of its 3 domains evolve rapidly to evade the viruses. Few residues within the dimerisation region of PKR have been shown to undergo positive selection [39]. This could mean that these could be NS5A interacting residues in PKR that are under positive selection in order to overcome the interaction with the viral protein NS5A. This shows the “Evolutionary arms race” [39] between the host protein PKR and the viral protein NS5A for their survival.

The information of interface residues in NS5A 1b and PKR would be very helpful to design inhibitors that would prevent this interaction to enable the HCV genotype 1 infected patients respond well to the interferon therapy like the other genotype infected patients of HCV.

## Methods

### Homology modeling of human casein kinase 1α

Human ck1α sequence [NCBI: NP_001020276.1] was retrieved from NCBI database. It was searched for similar sequences with known crystal structure in the protein databank by using BLAST program [47]. The homology model has been generated on the basis of the crystal structure of ck1α from *Schizosaccharomyces pombe* using COMPOSER [48,49], which has been incorporated in Sybyl (Tripos Inc. St Louis). Structurally conserved regions in the template structure are extrapolated to ck1α sequence. Template matching approach has been used for other regions which show sequence divergence from template sequence. These regions are modeled by finding a suitable fragment from a dataset which contains non identical protein structures using COMPOSER [50,51]. An insertion region (146–182) of human ck1α sequence [NCBI: NP_001020276.1) has not been modeled due to potentially poor reliability of the modeled conformation as there is no corresponding region in the template structure. This region is situated far away from the functional site of the kinase and so this region is not expected to influence the function of the kinase.

### Modeling of NS5A peptide bound to human casein kinase 1α model

NS5A peptide S(P)SAS has been modeled on to the structural model of ck1α which is re-generated based on the crystal structure of cAMP dependent kinase [PDB id: 1ATP] [19]. This is because firstly, the modeled CK1α adopts a similar fold with cAMP dependent protein kinase and the critical residues of both the kinases are topologically equivalent. Secondly, the substrate of cAMP dependent kinase has a substrate constraint at the third residue upstream of phosphoacceptor serine like ck1α. Hence ck1α was superposed on to cAMP dependent protein kinase- peptide complex by using the pairwise structure alignment program DALI [36]. Further, local superposition of critical regions like the glycine loop, catalytic loop in cAMP dependent kinase and ck1α model, were carried out using the program SUPER (B.S. Neela, unpublished). Therefore a model of ck1α bound to peptide inhibitor of cAMP dependent kinase at its substrate binding site was generated. The substrate peptide of cAMP dependent kinase has a phosphoacceptor Ser 21 which is replaced by alanine in the pseudo substrate, so that phosphorylation and dissociation of the peptide are not possible and the peptide is able to bind to the kinase. The substrate constraint residue which is three residues upstream to Ala 21 is a positively charged residue, Arginine [19]. This substrate recognition motif of cAMP dependent kinase is now replaced by those found in the NS5A substrate peptide (S (P) SAS) using Sybyl. The backbone of NS5A peptide is in the same conformation of the substrate recognition motif of cAMP dependent kinase. It was ensured that there were no short contacts in the ternary complex model of ck1α bound to HCV NS5A peptide along with ATP and was finally subjected to energy minimization using Kollman united atom model as the force field [52]. During energy minimization a distance criterion was imposed between the phosphoacceptor serine in the substrate and the catalytic base aspartic acid in the kinase, so that they are in proximity for the hydroxyl group of serine to accept the γ phosphate from ATP. It was ensured that the substrate interacting residues of the kinase and the substrate constraint residue phosphoserine are at optimal interacting distance. SYBYL and molecular visualization software PyMOL [33] were used for modeling and structural analysis of the complex.

### Modeling of p53 transactivation domain bound to human casein kinase 1α

A model of p53 transactivation domain for which the crystal structure is available containing the phosphoacceptor serine bound to ck1α model was generated as discussed in the previous section. It was made sure that the structure of p53 transactivation domain does not have any short contact with the kinase and makes favourable interactions with the residues of the kinase.

### Multiple sequence alignment of the members of casein kinase 1 family

Multiple sequence alignment of the members of casein kinase 1 family (from http://www.kinase.com/kinbase) was carried out using CLUSTALW [53]. The multiple sequence alignment was used to find if the substrate interacting residues in human casein kinase 1α was conserved across the casein kinase 1 family.

### Information derived from crystal structure of PKR dimer and alignment between NS5A and PKR

Crystal structure of PKR dimer was used to identify the interfacial residues in the PKR dimer [PDB id: 2A19] [19]. An atom pair across the PKR subunits having a distance less than the sum of the van der Waals radius of the 2 atoms + 0.5 Å has been considered to be interfacial residues [54,55].

Pairwise consensus sequence alignment were obtained from MAFFT, PROBCONS, T-coffee softwares [56-58] between the region in PKR that interacts with NS5A (PKR dimerisation region) and the ISDR in HCV NS5A protein (HCV-j 1b reference sequence). PKR dimer interface residues are noted in the PKR sequence and their equivalent residues in NS5A in the alignment reveals the putative residues in NS5A that could interact with PKR.

Multiple sequence alignment of NS5A sequences from the different genotypes was carried out using MAFFT [56]. The extent of conservation of the PKR interacting residues in NS5A 1b and equivalent residues from genotype 3a and 2a obtained from the multiple sequence alignment were calculated. The multiple sequence alignments were visualized using jalview [59].

### Evolutionary trace analysis to identify PKR interacting residues in NS5A 1b

#### Dataset

HCV polyprotein sequences were downloaded from European HCV database (735 sequences) [60]. The reference NS5A sequence [AF0096061a] was searched against the database containing 735 polyprotein HCV sequences. NS5A sequences were extracted from the 735 polyprotein sequences. Genotypes 1b, 2a and 3a sequences were only considered for the study because the clinical studies of response to interferon therapy, molecular studies of NS5A-PKR interaction and its relation to inhibition of PKR has been experimentally studied for these genotypes [14,29,30,40]. The strains used in the experimental studies and the reference strains of NS5A from genotype 1b, 2a and 3a were also added to the sequences for the analysis. Sequences with ambiguous amino acids (X) were omitted and only full length NS5A sequences were considered for the analysis. Since the sequences were highly similar they were clustered at 95% sequence identity using CD-HIT program [61] to get 69, 13, 7 representative sequences respectively in genotype 1b, 2a and 3a respectively.

#### Evolutionary trace analysis

In this study, trace residues have been defined as those residues which show a distinct sequence pattern in a particular position of both the multiple sequence alignment which is compared. Trace residues are picked up if complete/ high conservation (mostly >90% and very few residues showing >80%) / conservative substitution of two different residues or a residue and a gap from both multiple sequence alignment are being compared.

Seven representative sequences each from genotype 1b, 2a & 3a were chosen for the evolutionary trace analysis [31,32]. The multiple sequence alignment for the entire dataset of genotype 1b (331), 2a (16) and 3a (31) was carried out using MAFFT [56] and the extent of conservation of trace residues was calculated in percentage. The multiple sequence alignments were visualized and analyzed using jalview [59].

## Competing interests

The authors declared that they have no competing interests.

## Authors’ contributions

GS carried out the analysis. GS, YD and NT carried out the protein structure modeling. NS and SD conceived the study. All authors read and approved the final manuscript.

## Supplementary Material

Additional file 1 **Multiple sequence alignment of members among casein kinase 1 family.** The additional file provides multiple sequence alignment and the details of substrate interacting residues in human ck1α conserved within casein kinase 1 family. Arginine 214, lysine 260 is absolutely conserved and conservatively substituted respectively. Glycine 251 is not conserved because the main chain nitrogen is involved in its interaction with the substrate constraint residue – phosphoserine 229. These substrate interacting residues are highlighted in blue. The aspartic acid 140 of human ck1α probably avoids the proximity of substrate constraint acidic residue phosphoserine 229 of NS5A. Aspartic acid is conservatively substituted among the ck1 family and is highlighted in yellow. Click here for file

Additional file 2 **Prediction of PKR interacting residues in NS5A 1b.** (1) Alignment of PKR dimerisation region with ISDR (Interferon sensitivity determining region) of NS5A. (a) Dimer interface residues are colored in magenta in PKR sequence. The residues in NS5A 1b, 3a equivalent to the dimer interface residues of PKR are colored in cyan and yellow respectively. (b) Dimer interface residues are colored in magenta in PKR sequence. The residues in NS5A 1b, 2a equivalent to the dimer interface residues of PKR are colored in cyan and green respectively. (2) Trace residues are identified when NS5A from genotype 1b which interacts and inhibits PKR is compared with NS5A 3a and NS5A 2a which are unable to bind and inhibit PKR efficiently in few representative sequences. All the trace residues highlighted are highly conserved (> 80%) in the entire dataset. (a) Trace residues picked when MSA of genotype 1b Vs 3a is compared. (b) Trace residues picked when MSA of genotype 1b Vs 2a is compared. (c) Trace residues picked when MSA of genotype 1b Vs 3a and 2a are compared. Click here for file
