# Supplementary material for: Structural and molecular basis of interaction of HCV non-structural protein 5A with human casein kinase 1α and PKR
Source: BMC Struct Biol. 2012 Nov 13;12:28. doi: 10.1186/1472-6807-12-28 (PMC3534215; doi:10.1186/1472-6807-12-28)
Supplement: Additional file 1 — Multiple sequence alignment of members among casein kinase 1 family. The additional file provides multiple sequence alignment and the details of substrate interacting residues in human ck1α conserved within casein kinase 1 family. Arginine 214, lysine 260 is absolutely conserved and conservatively substituted respectively. Glycine 251 is not conserved because the main chain nitrogen is involved in its interaction with the substrate constraint residue – phosphoserine 229. These substrate interacting residues are highlighted in blue. The aspartic acid 140 of human ck1α probably avoids the proximity of substrate constraint acidic residue phosphoserine 229 of NS5A. Aspartic acid is conservatively substituted among the ck1 family and is highlighted in yellow. [file 1472-6807-12-28-S1.pdf]

```
CK1d_Hs         YRLGRKIGSGSFGDIYLGTDIAA-----------------GEEVAIKLECVKTKHPQLHI
CK1d_Mm         YRLGRKIGSGSFGDIYLGTDIAA-----------------GEEVAIKLECVKTKHPQLHI
CK1e_Hs         YRLGRKIGSGSFGDIYLGANIAS-----------------GEEVAIKLECVKTKHPQLHI
CK1e_Mm         YRLGRKIGSGSFGDIYLGANIAS-----------------GEEVAIKLECVKTKHPQLHI
AqueK089_Aq     YRLGRKIGSGSFGDIYLGTNIAT-----------------KEEVAIKLECVKTKHPQLHI
CK1d_Sp         YRLGRKIGSGSFGDIYLGTNITN-----------------GEEVAIKLECLKTKHPQLHI
dco_Dm          YRLGRKIGSGSFGDIYLGTTINT-----------------GEEVAIKLECIRTKHPQLHI
F46F2.2_Ce      FRLGRKIGSGSFGDIYLGQNIQT-----------------NEEVAVKLECVKSKHPQLHI
CK1_Dd          YRISRKIGGGSFGEIYLGTNIST-----------------NEEVAIKLEPAKAIHPQLLF
CK1_C2d_Dd      YRISRKIGGGSFGEIYLGTNIST-----------------NEEVAIKLEPAKAIHPQLLF
HRR25_Sc        FRIGRKIGSGSFGDIYHGTNLIS-----------------GEEVAIKLESIRSRHPQLDY
CK1g1_Hs        FRVGKKIGCGNFGELRLGKNLYT-----------------NEYVAIKLEPIKSRAPQLHL
CK1g1_Mm        FRVGKKIGCGNFGELRLGKNLYT-----------------NEYVAIKLEPIKSRAPQLHL
CK1g2_Hs        FRVGKKIGCGNFGELRLGKNLYT-----------------NEYVAIKLEPIKSRAPQLHL
CK1g2_Mm        FRVGKKIGCGNFGELRLGKNLYT-----------------NEYVAIKLEPIKSRAPQLHL
CK1g3_Hs        FRVGKKIGCGNFGELRLGKNLYT-----------------NEYVAIKLEPMKSRAPQLHL
CK1g3_Mm        FRVGKKIGCGNFGELRLGKNLYT-----------------NEYVAIKLEPMKSRAPQLHL
AqueK090_Aq     FRVGKKIGCGNFGELRLGKNLYN-----------------NEHVAIKLEPMKSRAPQLHL
gish_Dm         FRVGKKIGCGNFGELRLGKNLYN-----------------NEHVAIKMEPMKSKAPQLHL
CK1g_Sp         FRVGKKIGCGNFGELRLGKNLYN-----------------NEHVAIKLEPMRSRAPQLHL
Y106G6E.6_Ce    FKVGKKIGCGNFGELRLGKNLYN-----------------NEHVAIKLEPMKSKAPQLHL
YCK1_Sc         YKIGKKIGEGSFGVLFEGTNMIN-----------------GVPVAIKFEPRKTEAPQLRD
YCK2_Sc         YKIGKKIGEGSFGVLFEGTNMIN-----------------GLPVAIKFEPRKTEAPQLKD
YCK3_Sc         YAVGPKIGEGSFGVIFEGENILHSCQAQTGSKRDSSIIMANEPVAIKFEPRHSDAPQLRD
GK019_Gl        YQLHYRIGRGAFGELYRGVNLAT-----------------GEDVAIKLEPIDAKPPQLLY
CK1a_Hs         YKLVRKIGSGSFGDIYLAINITN-----------------GEEVAVKLESQKARHPQLLY
CK1a_Mm         YKLVRKIGSGSFGDIYLAINITN-----------------GEEVAVKLESQKARHPQLLY
CK1a2_Hs        YKLVRKIGSGSFGDVYLGITTTN-----------------GEDVAVKLESQKVKHPQLLY
AqueK088_Aq     YRLVRKIGSGSFGDIYLGVNQTN-----------------GEEVAVKLESHKARHPQLLY
C03C10.1_Ce     YKLIRKIGSGSFGDIYVSINVTN-----------------GEEVAIKLESNRARHPQLLY
CK1a2_Sp        YKLVRKIGSGSFGDIYLGINVGN-----------------GEEIAVKMESAKARHPQLNY
CK1a1_Sp        YRLVRKIGSGSFGDIYLGINIHN-----------------GEEVAVKLESEKARHPQLLY
CkIalpha_Dm     YRVIRKIGSGSFGDIYLGMSIQS-----------------GEEVAIKMESAHARHPQLLY
CG7094_Dm       YRLVKPIGSGSFGDIYLGLSITD-----------------GSEVAIKVEKNDAKYPQLIY
CG2577_Dm       YKVVRKIGCGSFGDIYLGIYIHS-----------------GERVAIKVESSKVRHPQLNY
CG12147_Dm      YRLLKRIGNGSFGELFQAEGLKY-----------------HEKVAIKLESSTVKHPLLPR
CG9962_Dm       IMVIRKLGSGSFGDIYEAKHMGS-----------------GLHVALKVERKNAGQSHLSI
                  :   :* * ** :  .                         :*:*.*      . *

CK1d_Hs         ESKIYKMMQGGV------------------------------GIPTIRWCGAEGDYNVMV
CK1d_Mm         ESKIYKMMQGGV------------------------------GIPTIRWCGAEGDYNVMV
CK1e_Hs         ESKFYKMMQGGV------------------------------GIPSIKWCGAEGDYNVMV
CK1e_Mm         ESKFYKMMQGGV------------------------------GIPSIKWCGAEGDYNVMV
AqueK089_Aq     EAKFYKMMQSGV------------------------------GIPSIKWCGTEGDYNVLV
CK1d_Sp         ESKFYKMMQGGV------------------------------GIPTVKWCGAEGDYNVMV
dco_Dm          ESKFYKTMQGGI------------------------------GIPRIIWCGSEGDYNVMV
F46F2.2_Ce      ESRLYRIMLGGI------------------------------GIPEIRWCGQEGDYNVMV
CK1_Dd          ESKLYKIFQGGI------------------------------GIPAVKWFGFDGDYNIMV
CK1_C2d_Dd      ESKLYKIFQGGI------------------------------GIPAVKWFGFDGDYNIMV
HRR25_Sc        ESRVYRYLSGGV------------------------------GIPFIRWFGREGEYNAMV
CK1g1_Hs        EYRFYKQLGSAG-----------------------------EGLPQVYYFGPCGKYNAMV
CK1g1_Mm        EYRFYKQLGSAG-----------------------------EGLPQVYYFGPCGKYNAMV
CK1g2_Hs        EYRFYKQLSATGTGRPAGGAGAAQGQGGGCRRTPVRLSSAAEGVPQVYYFGPCGKYNAMV
CK1g2_Mm        EYRFYKQLSTTGEADSGTGP-ALLGQQ--WLRTPSMDVSFAEGVPQVYYFGPCGKYNAMV
CK1g3_Hs        EYRFYKQLGSG------------------------------DGIPQVYYFGPCGKYNAMV
CK1g3_Mm        EYRFYKQLGSG------------------------------DGIPQVYYFGPCGKYNAMV
AqueK090_Aq     EYRFYRTLGNA------------------------------VGIPQVYYFGPCGKYNAMV
gish_Dm         EYRFYKLLGSHA-----------------------------EGVPEVYYFGPCGKYNALV
CK1g_Sp         EYRFYKMLG-QS-----------------------------EGIPQVHYFGPCGKYNAMV
Y106G6E.6_Ce    EYRFYKLLG-QA-----------------------------EGLPQVHYFGPCGKYNALV
YCK1_Sc         EYKTYKILNGTP------------------------------NIPYAYYFGQEGLHNILV
YCK2_Sc         EYRTYKILAGTP------------------------------GIPQEYYFGQEGLHNILV
YCK3_Sc         EFRAYRILNGCV------------------------------GIPHAYYFGQEGMHNILI
GK019_Gl        ESKLYKILVGGV------------------------------GIPYIYHYAPEGEYNVLV
CK1a_Hs         ESKLYKILQGGV------------------------------GIPHIRWYGQEKDYNVLV
CK1a_Mm         ESKLYKILQGGV------------------------------GIPHIRWYGQEKDYNVLV
CK1a2_Hs        ESKLYTILQGGV------------------------------GIPHMHWYGQEKDNNVLV
AqueK088_Aq     ESKLYKILQGGV------------------------------GIPHIKWYGSEKEYNVLV
C03C10.1_Ce     ESKVYRILQGGV------------------------------GIPHIRWYGTEREYNVLV
CK1a2_Sp        EGKVYRILQGGV------------------------------GIPKIRWYGSEKDYNVLV
CK1a1_Sp        ESKLYKILHGGP------------------------------GIPTMKWFGQGHDYNIMV
```

```
CkIalpha_Dm        EAKLYRILSGGV------------------------------GFPRIRHHGKEKNFNTLV
CG7094_Dm          EAKVYEQLARCP------------------------------GFPTLLHYGCEKNYNAMV
CG2577_Dm          ERRIYRALRPAH------------------------------GLPRIRYFHKEEHYQAMV
CG12147_Dm         EARIYGILQGGL------------------------------GIPHVKHYATEGAYNVMV
CG9962_Dm          ESTVYNLLRHGM------------------------------GIPMTYQFFSNRRHDVLV
                   *   *  :                                  ..*           : ::

CK1d_Hs            MELLGPSLEDLFNFCSRKFSLKTVLLLADQMISRIEYIHSKNFIHRDVKPDNFLMGL---
CK1d_Mm            MELLGPSLEDLFNFCSRKFSLKTVLLLADQMISRIEYIHSKNFIHRDVKPDNFLMGL---
CK1e_Hs            MELLGPSLEDLFNFCSRKFSLKTVLLLADQMISRIEYIHSKNFIHRDVKPDNFLMGL---
CK1e_Mm            MELLGPSLEDLFNFCSRKFSLKTVLLLADQMISRIEYIHSKNFIHRDVKPDNFLMGL---
AqueK089_Aq        MELLGPSLEDLFNFCSRKFSLKTVLLLADQLINRIEYIHSKNFIHRDVKPDNFLMGL---
CK1d_Sp            MELLGPSLEDLFNFCSREFSLKTVLLLADQLISRIEYVHSKNFIHRDIKPDNFLMGL---
dco_Dm             MELLGPSLEDLFNFCSRRFSLKTVLLLADQMISRIDYIHSRDFIHRDIKPDNFLMGL---
F46F2.2_Ce         MELLGPSLEDLFNFCQRKFSLKTVLLLADQMLSRVEFIHCRDYIHRDIKPDNFLMGL---
CK1_Dd             MDLLGPSLEDLFNYCGRKFSLKTVLMLGDQMLRRIEFIHSNNFIHRDIKPDNFLMGI---
CK1_C2d_Dd         MDLLGPSLEDLFNYCGRKFSLKTVLMLGDQMLRRIEFIHSNNFIHRDIKPDNFLMGI---
HRR25_Sc           IDLLGPSLEDLFNYCHRRFSFKTVIMLALQMFCRIQYIHGRSFIHRDIKPDNFLMGV---
CK1g1_Hs           LELLGPSLEDLFDLCDRTFTLKTVLMIAIQLLSRMEYVHSKNLIYRDVKPENFLIGR---
CK1g1_Mm           LELLGPSLEDLFDLCDRTFTLKTVLMIAIQLLSRMEYVHSKNLIYRDVKPENFLIGR---
CK1g2_Hs           LELLGPSLEDLFDLCDRTFTLKTVLMIAIQLITRMEYVHTKSLIYRDVKPENFLVGR---
CK1g2_Mm           LELLGPSLEDLFDLCDRTFTLKTVLMIAIQLITRMEYVHTKSLIYRDVKPENFLVGR---
CK1g3_Hs           LELLGPSLEDLFDLCDRTFSLKTVLMIAIQLISRMEYVHSKNLIYRDVKPENFLIGR---
CK1g3_Mm           LELLGPSLEDLFDLCDRTFSLKTVLMIAIQLISRMEYVHSKNLIYRDVKPENFLIGR---
AqueK090_Aq        LELLGPSLEDLFDLCNRKFSLKTVLMIAIQLITRIEYVHTKHLIYRDVKPENFLMGR---
gish_Dm            MELLGPSLEDLFDICGRRFTLKSVLLIAIQLLHRIEYVHSRHLIYRDVKPENFLIGR---
CK1g_Sp            MELLGPSLEDLFDLCNRQFSLKTVLLLSTQLLHRIEYVHSKHLIYRDVKPENFLIGR---
Y106G6E.6_Ce       MELLGHSLEDLFDLCDRHFSLKTVAMVAMQLIRRFEYVHTKHLFYGDVKPENFLIGR---
YCK1_Sc            IDLLGPSLEDLFDWCGRKFSVKTVVQVAVQMITLIEDLHAHDLIYRDIKPDNFLIGR---
YCK2_Sc            IDLLGPSLEDLFDWCGRRFSVKTVVQVAVQMITLIEDLHAHDLIYRDIKPDNFLIGR---
YCK3_Sc            IDLLGPSLEDLFEWCGRKFSVKTTCMVAKQMIDRVRAIHDHDLIYRDIKPDNFLISQYQR
GK019_Gl           MDLLGPSLEDLFTACNRHFSLKTVLMLADQMITRVEFLHLKNFLHRDIKPDNFLIGL---
CK1a_Hs            MDLLGPSLEDLFNFCSRRFTMKTVLMLADQMISRIEYVHTKNFIHRDIKPDNFLMGI---
CK1a_Mm            MDLLGPSLEDLFNFCSRRFTMKTVLMLADQMISRIEYVHTKNFIHRDIKPDNFLMGI---
CK1a2_Hs           MDLLGPSLEDLFNFCSRRFTMKTVLMLADQMISRIEYVHTKNFLHRDIKPDNFLMGT---
AqueK088_Aq        MDLLGPSLEDLFNFCSRRFTMKTVLMLADQMIGRIEYVHNKNFIHRDIKPDNFLMGI---
C03C10.1_Ce        MDLLGPSLEDLFNFCSRRFTMKTVLMLADQMIGRIEYVHVKNFIHRDIKPDNFLMGI---
CK1a2_Sp           MELLGPSLEDLFNFCDRQFSMKTVLMLADQMLGRIEYVHNKNFIHRDIKPDNFLMGI---
CK1a1_Sp           MELLGPSLEDLFNFCSRKFTMKTVLMLADQMIARIEYVHVKNFIHRDIKPDNFLMGI---
CkIalpha_Dm        MDLLGPSLEDLFNFCTRHFTIKTVLMLVDQMIGRLEYIHLKCFIHRDIKPDNFLMGI---
CG7094_Dm          MDLLGPSLEELFNLCKRRFSLKTVLMLTDQLLMRIECVHERGFIHRDIKPDNFLMGL---
CG2577_Dm          MDLLGPSLERLFQFCERAFTIKTVLLLAEQMLRRVEYVHNRGFLHRDIKPDNFLMGL---
CG12147_Dm         MDLLGPTLEDLLNLCSRSFSMKTTLMLADQILARVELLHRRCFIHRDIKPDNFLMGL---
CG9962_Dm          MELLGPSLETLFTMCNRRFSMKTVLMLADQMVDRLEYLHLHHYVHRDIKPENFLMGV---
                   ::*** :** *:  * * *:.*:.  :  *:.  .  :* .  .: *:**:***:.

CK1d_Hs            ---------------GKKGN----------------------------LVYIIDFGLAKK
CK1d_Mm            ---------------GKKGN----------------------------LVYIIDFGLAKK
CK1e_Hs            ---------------GKKGN----------------------------LVYIIDFGLAKK
CK1e_Mm            ---------------GKKGN----------------------------LVYIIDFGLAKK
AqueK089_Aq        ---------------GKKGN----------------------------LVYIIDFGLAKK
CK1d_Sp            ---------------GKKGN----------------------------MVYIIDFGLAKR
dco_Dm             ---------------GKKGN----------------------------LVYIIDFGLAKK
F46F2.2_Ce         ---------------GKRGN----------------------------LVYIIDFGLAKR
CK1_Dd             ---------------GKRGH----------------------------VVNLIDFGLAKR
CK1_C2d_Dd         ---------------GKRGH----------------------------VVNLIDFGLAKR
HRR25_Sc           ---------------GRRGS----------------------------TVHVIDFGLSKK
CK1g1_Hs           -------------QGNKKEH----------------------------VIHIIDFGLAKE
CK1g1_Mm           -------------QGNKKEH----------------------------VIHIIDFGLAKE
CK1g2_Hs           -------------PGTKRQH----------------------------AIHIIDFGLAKE
CK1g2_Mm           -------------PGSKRQH----------------------------SIHIIDFGLAKE
CK1g3_Hs           -------------PGNKTQQ----------------------------VIHIIDFGLAKE
CK1g3_Mm           -------------PGNKAQQ----------------------------VIHIIDFGLAKE
AqueK090_Aq        -------------YGTPNQN----------------------------IVNIIDFGLAKE
gish_Dm            -------------TSTKREK----------------------------IIHIIDFGLAKE
CK1g_Sp            -------------SSSKTQN----------------------------TIHIIDFGLAKE
Y106G6E.6_Ce       -------------YSTRKQH----------------------------VLHIIDFGLAKE
YCK1_Sc            -------------PGQPDAN----------------------------NIHLIDFGMAKQ
YCK2_Sc            -------------PGQPDAN----------------------------KVHLIDFGMAKQ
YCK3_Sc            ISPEGKVIKSCASSSNNDPN----------------------------LIYMVDFGMAKQ
GK019_Gl           ---------------GRRKT----------------------------TVYIIDFGLAKR
```

```
CK1a_Hs              ---------------GRHCNKCLESPVGKRKRSMTVSTSQDPSFSGLNQLFLIDFGLAKK
CK1a_Mm              ---------------GRHCNKCLESPVGKRKRSMTVSPSQDPSFSGLNQLFLIDFGLAKK
CK1a2_Hs             ---------------GRHCN----------------------------KLFLIDFGLAKK
AqueK088_Aq          ---------------GRHCN----------------------------KLFIIDFGLAKK
C03C10.1_Ce          ---------------GRHCN----------------------------KLFLIDFGLAKK
CK1a2_Sp             ---------------GRHCN----------------------------QVFIIDFGLAKK
CK1a1_Sp             ---------------GRHCN----------------------------QLFLIDFGLAKK
CkIalpha_Dm          ---------------GRHCN----------------------------KLFLIDFGLAKK
CG7094_Dm            ---------------DRHCN----------------------------KLYLIDFGLSKR
CG2577_Dm            ---------------GTMSK----------------------------QVYLIDFGLSKK
CG12147_Dm           ---------------NRHQT----------------------------QVYMIDFGLAKK
CG9962_Dm            ---------------GLTRH----------------------------RLHLIDFGLSKR
                                                                      : ::***::*.

CK1d_Hs              YRDARTHQHIPYRENKNLTGTARYASINTHLGIEQSRRDDLESLGYVLMYFNLGSLPWQG
CK1d_Mm              YRDARTHQHIPYRENKNLTGTARYASINTHLGIEQSRRDDLESLGYVLMYFNLGSLPWQG
CK1e_Hs              YRDARTHQHIPYRENKNLTGTARYASINTHLGIEQSRRDDLESLGYVLMYFNLGSLPWQG
CK1e_Mm              YRDARTHQHIPYRENKNLTGTARYASINTHLGIEQSRRDDLESLGYVLMYFNLGSLPWQG
AqueK089_Aq          YRDTRTHQHIPYRENKNLTGTARYASINTHLGIEQSRRDDLESLGYMLMYFNLGNLPWQG
CK1d_Sp              YKDPRTKLHIAYRENKNLTGTARYASINTHLGIEQSRRDDLESLGYMLMYFNRGSLPWQG
dco_Dm               FRDARSLKHIPYRENKNLTGTARYASINTHLGIEQSRRDDLESLGYVLMYFNLGALPWQG
F46F2.2_Ce           YRDSK-HQHIAYRENKNLTGTARYASINTHRGIEQSRRDDIESLGYVFMYFNRGTLPWQG
CK1_Dd               YRDPKTHQHIPYREHKNLTGTARYASLNTHQGIEQSRRDDLESLGYVLMYFNRGSLPWQG
CK1_C2d_Dd           YRDPKTHQHIPYREHKNLTGTARYASLNTHQGIEQSRRDDLESLGYVLMYFNRGSLPWQG
HRR25_Sc             YRDFNTHRHIPYRENKSLTGTARYASVNTHLGIEQSRRDDLESLGYVLIYFCKGSLPWQG
CK1g1_Hs             YIDPETKKHIPYREHKSLTGTARYMSINTHLGKEQSRRDDLEALGHMFMYFLRGSLPWQG
CK1g1_Mm             YVDPETKKHIPYREHKSLTGTARYMSINTHLGKEQSRRDDLEALGHMFMYFLRGSLPWQG
CK1g2_Hs             YIDPETKKHIPYREHKSLTGTARYMSINTHLGKEQSRRDDLEALGHMFMYFLRGSLPWQG
CK1g2_Mm             YIDPETKKHIPYREHKSLTGTARYMSINTHLGKEQSRRDDLEALGHMFMYFLRGSLPWQG
CK1g3_Hs             YIDPETKKHIPYREHKSLTGTARYMSINTHLGKEQSRRDDLEALGHMFMYFLRGSLPWQG
CK1g3_Mm             YIDPETKKHIPYREHKSLTGTARYMSINTHLGKEQSRRDDLEALGHMFMYFLRGSLPWQG
AqueK090_Aq          YIDPDTTKHIPYREHKSLTGTARYMSINTHLGKEQSRRDDLEALGHMFMYFLRGSLPWQG
gish_Dm              YIDLDTNRHIPYREHKSLTGTARYMSINTHMGREQSRRDDLEALGHMFMYFLRGSLPWQG
CK1g_Sp              YIDPETNKHIPYREHKSLTGTARYMSINTHLGKEQSRRDDLEALGHMFMYFLRGSLPWQG
Y106G6E.6_Ce         YIDCDTGKHIAYREHKSLTGTARYMSINTHLGKEQSRRDDLEALGHMFMYFLRGSLPWQG
YCK1_Sc              YRDPKTKQHIPYREKKSLSGTARYMSINTHLGREQSRRDDMEALGHVFFYFLRGHLPWQG
YCK2_Sc              YRDPKTKQHIPYREKKSLSGTARYMSINTHLGREQSRRDDMEAMGHVFFYFLRGQLPWQG
YCK3_Sc              YRDPRTKQHIPYRERKSLSGTARYMSINTHFGREQSRRDDLESLGHVFFYFLRGSLPWQG
GK019_Gl             YMVGNT--HIPYREHKSLTGTARYCSINTHLGLEQSRRDDLEALAYVFLYFLRGFLPWQG
CK1a_Hs              YRDNRTRQHIPYREDKNLTGTARYASINAHLGIEQSRRDDMESLGYVLMYFNRTSLPWQG
CK1a_Mm              YRDNRTRQHIPYREDKNLTGTARYASINAHLGIEQSRRDDMESLGYVLMYFNRTSLPWQG
CK1a2_Hs             YRDNRTRQHIPYREDKHLIGTVRYASINAHLGIEQSRRDDMESLGYVFMYFNRTSLPWQG
AqueK088_Aq          YRDTRSKQHIPYREDKNLTGTARYASINAHLGIEQSYR----------------------
C03C10.1_Ce          YRDSRTRTHIPYREDKNLTGTARYASINAHLGIEQSRRDDMESLGYVLMYFNRGTLPWQG
CK1a2_Sp             YRDSRTRQHIPYKDDKNLTGTARYASIHAHQGIEQARRDDLESLGYVMMYFNRRSLPWQG
CK1a1_Sp             FKDSRSKQHIQYREDKNLTGTARYASINAHLGIEQSRRDDLESLGYVLMYFNRTSLPWQG
CkIalpha_Dm          FRDPHTRHHIVYREDKNLTGTARYASINAHLGIEQSRRDDMESLGYVMMYFNRGVLPWQG
CG7094_Dm            YKDIESEIHIPYRTDRNLTGTVRYASINAQIGVEQSRRDDMESMSYCLMYFNLGKLPWQG
CG2577_Dm            YLDITTGVHIPYREERSLTGTARYASIGAHAGVESARRDDMVAVGYVLMYFNRGSLPWQD
CG12147_Dm           FYSLRTQKHIGYTENRDLVGTARYASVRAHY-AEQSRRDDLESVGYLLLYFQRGRLPWQG
CG9962_Dm            YWDMKENRHVPQRRGTKWAGTARYASVNALCCKVQSRRDDLESVGYVLIYLLRGSLPWQG
                     :       *:         **.** *: :     .: *

CK1d_Hs              LKAATKRQKYERISEKKMSTPIEVLCKG--YPSEFATYLNFCRSLRFDDKPDYSYL----
CK1d_Mm              LKAATKRQKYERISEKKMSTPIEVLCKG--YPSEFATYLNFCRSLRFDDKPDYSYL----
CK1e_Hs              LKAATKRQKYERISEKKMSTPIEVLCKG--YPSEFSTYLNFCRSLRFDDKPDYSYL----
CK1e_Mm              LKAATKRQKYERISEKKMSTPIEVLCKG--YPSEFSTYLNFCRSLRFDDKPDYSYL----
AqueK089_Aq          LKAATKKQKYEKISEKKLSTPVE-------------------------------------
CK1d_Sp              LKAATKKQKYEKISEKKMSTP---------------------------------------
dco_Dm               LKAANKRQKYERISEKKLSTSIVVLCKG--FPSEFVNYLNFCRQMHFDQRPDYCHLRKLF
F46F2.2_Ce           LKAVTKRQKYELISEKKISTRVDDLCAG--YPEAFAQYLNY-------------------
CK1_Dd               LKAYTKRDKYEKICDKKAQTKIDTLCQG--FPSEFATFLNYTRFLKFEDKPDFLYL----
CK1_C2d_Dd           LKAYTKRDKYEKICDKKAQTKIDTLCQG--FPSEFATFLNYTRFLKFEDKPDFLYL----
HRR25_Sc             LKATTKKQKYDRIMEKKLNVSVETLCSG--LPLEFQEYMAYCKNLKFDEKPDYL------
CK1g1_Hs             LKADTLKERYQKIGDTKRNTPIEALCEN--FPEEMATYLRYVRRLDFFEKPDYEYL----
CK1g1_Mm             LKADTLKERYQKIGDTKRNTPIEALCEN--FPEEMATYLRYVRRLDFFEKPDYEYL----
CK1g2_Hs             LKADTLKERYQKIGDTKRATPIEVLCEN--FPEEMATYLRYVRRLDFFEKPDYDYL----
CK1g2_Mm             LKADTLKERYQKIGDTKRATPIEVLCES--FPEEMATYLRYVRRLDFFEKPDYDYL----
CK1g3_Hs             LKADTLKERYQKIGDTKRATPIEVLCEN--FP-EMATYLRYVRRLDFFEKPDYDYL----
CK1g3_Mm             LKADTLKERYQKIGDTKRATPIEVLCEN--FPEEMATYLRYVRRLDFFEKPDYDYL----
AqueK090_Aq          LKADTLKERYQKIGETKRSTPIE-------------------------------------
```

```
gish_Dm           LKADTLKERYQKIGDTKRATPIEVLCDG--HPEEFATYLRYVRRLDFFETPDYDFLRRLF
CK1g_Sp           LKADTLKER---------------------------------------------------
Y106G6E.6_Ce      LKADTLKERYQKIGDTKRQTAVEVLCEG--FPDEFAQYLRYARRLDFF------------
YCK1_Sc           LKAPNNKQKYEKIGEKKRSTNVYDLAQG--LPVQFGRYLEIVRSLSFEECPDYE------
YCK2_Sc           LKAPNNKQKYEKIGEKKRLTNVYDLAQG--LPIQFGRYLEIVRNLSFEETPDYE------
YCK3_Sc           LKAPNNKLKYEKIGMTKQKLNPDDLLLNNAIPYQFATYLKYARSLKFDEDPDYD------
GK019_Gl          LKANTKRQKYEKILEKKLSSPIES------------------------------------
CK1a_Hs           LKAATKKQKYEKISEKKMSTPVEVLCKG--FPAEFAMYLNYCRGLRFEEAPDYMYL----
CK1a_Mm           LKAATKKQKYEKISEKKMSTPVEVLCKG--FPAEFAMYLNYCRGLRFEEAPDYMYL----
CK1a2_Hs          LRAMTKKQKYEKISEKKMSTPVEVLCKG--FPAEFAMYLNYCRGLRFEEVPDYMYL----
AqueK088_Aq       ------------------------------------------------------------
C03C10.1_Ce       LKAATKKQKYEKISEKKMTTSVEHLCKG--FPAEFPMYLSYTRGLRFDESPDYMYLRQLF
CK1a2_Sp          LKAATKKQKYEKISEKKM------------------------------------------
CK1a1_Sp          MKAMTKKQKYEKISEKKMSTP---------------------------------------
CkIalpha_Dm       MKANTKQQKYEKISEKKMSTPIEVLCKG--SPAEFSMYLNYCRSLRFEEQPDYMYLRQLF
CG7094_Dm         ITAANKKQKYEKILEKKTSVTIAQLCKG--FPSEFCLLMTYVRNLGFKEPPDHTYLRQIF
CG2577_Dm         LKASTKQQKYERIHEKKISVSIEVLCEG--FPCEFTMYLNYCRGMGFYDKPNYDFICRMF
CG12147_Dm        IRAQSQAQKYEKIAEYKANIPLQQLCSG--LPVEFFMYLKYCRKLHFAEKPDYV------
CG9962_Dm         LLPNSKLQKAEMILEMKLSTLPNSLCAG--YPNEFYNYIIYTRQLGFEEEPDYRMIRCTF


CK1d_Hs           ------------------
CK1d_Mm           ------------------
CK1e_Hs           ------------------
CK1e_Mm           ------------------
AqueK089_Aq       ------------------
CK1d_Sp           ------------------
dco_Dm            ------------------
F46F2.2_Ce        ------------------
CK1_Dd            ------------------
CK1_C2d_Dd        ------------------
HRR25_Sc          ------------------
CK1g1_Hs          ------------------
CK1g1_Mm          ------------------
CK1g2_Hs          ------------------
CK1g2_Mm          ------------------
CK1g3_Hs          ------------------
CK1g3_Mm          ------------------
AqueK090_Aq       ------------------
gish_Dm           QDLFDRKGYTDEGEF---
CK1g_Sp           ------------------
Y106G6E.6_Ce      ------------------
YCK1_Sc           ------------------
YCK2_Sc           ------------------
YCK3_Sc           ------------------
GK019_Gl          ------------------
CK1a_Hs           ------------------
CK1a_Mm           ------------------
CK1a2_Hs          ------------------
AqueK088_Aq       ------------------
C03C10.1_Ce       RILFRTLNHQYDYTFDWT
CK1a2_Sp          ------------------
CK1a1_Sp          ------------------
CkIalpha_Dm       RILFRTLNHQYDYI----
CG7094_Dm         RILFRSLNHHYDYI----
CG2577_Dm         RMLRNGLNL---------
CG12147_Dm        ------------------
CG9962_Dm         LSLLFNLKFTNDLIYDWD
```
