# Supplementary material for: Structural and molecular basis of interaction of HCV non-structural protein 5A with human casein kinase 1α and PKR
Source: BMC Struct Biol. 2012 Nov 13;12:28. doi: 10.1186/1472-6807-12-28 (PMC3534215; doi:10.1186/1472-6807-12-28)
Supplement: Additional file 2 — Prediction of PKR interacting residues in NS5A 1b. (1) Alignment of PKR dimerisation region with ISDR (Interferon sensitivity determining region) of NS5A. (a) Dimer interface residues are colored in magenta in PKR sequence. The residues in NS5A 1b, 3a equivalent to the dimer interface residues of PKR are colored in cyan and yellow respectively. (b) Dimer interface residues are colored in magenta in PKR sequence. The residues in NS5A 1b, 2a equivalent to the dimer interface residues of PKR are colored in cyan and green respectively. (2) Trace residues are identified when NS5A from genotype 1b which interacts and inhibits PKR is compared with NS5A 3a and NS5A 2a which are unable to bind and inhibit PKR efficiently in few representative sequences. All the trace residues highlighted are highly conserved (> 80%) in the entire dataset. (a) Trace residues picked when MSA of genotype 1b Vs 3a is compared. (b) Trace residues picked when MSA of genotype 1b Vs 2a is compared. (c) Trace residues picked when MSA of genotype 1b Vs 3a and 2a are compared. [file 1472-6807-12-28-S2.pdf]

```
(1a)            HPDAELVDANLLWRQEMGSNI---TRVESETKVVILDSFEPLRAETDDNELSVAAEC
                HPDAELVDANLLWRQEMGSNI---TRVESETKVVILDSFEPLRAETDDVELSVAAEC
                HPDAELVDANLLWRQEMGSNI---TRVESESKVVILDSFEPLRAEPDDAELSVAAEC
                HPDAELVDANLLWRQEMGSNI---TRVESESKVVILDSFEPLRAEPDDAELSVAAEC
                HPDAELVDANLLWRQEMGSNI---TRVESETKVVILDSFEPLRAEADDAELSVAAEC
                HPDAELVDANLLWRQEMGSNI---TRVESETKVVILDSFEPLRAEDDDAELSVAAEC
                HPDAELVDANLLWRQEMGSNI---TRVESETKVVILDSFEPLRAETDDAELSVAAEC
                HPDAELVDANLLWRQEMGSNI---TRVESETKVVILDSFEPLRAETDDAELSVAAEC
                HPDAELVDANLLWRQEMGSNI---TRVESETKVVILDSFEPLRAETDDAELSVAAEC
 Genotype 3a    HPDAELVDANLLWRQEMGSNI---TRVESETKVVILDSFEPLRAETDDAELSVAAEC
                HPDAELVDANLLWRQEMGSNI---TRVESETKVVVLDSFEPLRAETDDVEPSVAAEC
                HPDAELVNANLLWRQEMGSNI---TRVESETKVVILDSFEPLRAETDDAELSVAAEC
                HPDAELVDANLLWRQEMGSNI---TRVESETKVVILDSFEPLRAETDDAELSVAAEC
                HPDAELVDANLLWRQEMGSNI---TRVESETKVVILDSFEPLRAQTDDAELSVAAEC
                HPDAELVDANLLWRQEMGSDI---TRVESKTKVVILDSFEPLRAEDDDAELSVAAEC
                HPDVELVDANLLWRQEMGSNI---TRVESETKVVILDSFEPLRAEPDDIELSVAAEC
                HPDAELVDANLLWRQEMGSNI---TRVESETKVVILDSFEPLRAETDDVELSVAAEC
                HPDAELVDANLLWRQEMGSNI---TRVESETKVVILDSFEPLRAETDDTELSVAAEC
                HPDAELVDANLLWRQEMGSNI---TRVESETKVVILDSFEPLKAETDDIELSVAAEC
                HPDAELVDANLLWRQEMGSNI---TRVESETKVVILDSFEPLRAETDDAELSAAAEC
                HPDAELVDANLLWRQEMGSNI---TRVESETKVVILDSFEPLRAETDDAELSVAAEC
                HPDAELVDANLLWRQEMGSNI---TRVESETKVVILDSFEPLRAEPDDTELSVAAEC
                HPDAELVDANLLWRQEMGSNI---TRVESETKVVILDSFEPLKAETDDTELSVAAEC
                YPDAELVDANLLWRQEMGSNI---TRVESETKVVILDSFEPLRAETDDAELSVAAEC
                HPDAELVDANLLWRQEMGSYI---TRLESETEVVVLDSFEPLRAETDDTELSVTAEC

                SPDVDLIEANLLWQQVMGGNI---TRVESENKVVILDSFVPLRAEEDEREISTPAEI
 Genotype 1b    SPDADLIEANLLWQQEMGGNI---TRVESENKVVILDSFEPLRAEEGEREVSVPAEI
                PPDIDLIEANLLWRQEMGGNI---TRVESENKVVILDSFDPLRTEEDEGEISVAAEI
                SPDVDLIEANLLWRQEMGGNI---TRVESENKVVVLDSFEPLRAEGDENEISIAAEI
                SPDADLIEANLLWRQEMGGNI---TRVESENKVVILDSFDPLRAEEDEREVSVAAEI
                SPDTDLVEANLMWRQVMGGSI---TRVESENKVVILDSFVPLRTEEDEREVSVPAEI
                SPDVDLLEANLLWQQEMGGNI---TRVESEKKVVILDSFEPLRAEEDEREMSVPAEI
                SPDADLIEANLLWRQEMGGNI---TRVESENKVVILDSFDPIRAVEDEREISVPAEI
   PKR          RFGMDFKEIELIGSGGFGQVFKAKHRIDGKTYVIKRVKYNNE---KAEREVKALAKL


(1b)
                TYDVDMVDANLL----MEGGV---AQTEPESRVPVLDFLEPMAEEE-SDLEPSIPSE
                AYDVDMVDANLF----MGGDV---TRIESESKVVVLDSLDPMVEER-SDLEPSIPSE
                AYDVDMVDANLF----MGGDV---TRIESGSKVVVLDSLDPMVEER-SDLEPSIPSE
                THDVDMVDANLF----MGGDV---TRIESESKVVVLDSLDPMVEEE-GDLEPSIPSE
                TYDVDMVDASLF----MGGDV---TRIESESKVVVLDSLDPMAEEE-SDLEPSIPSE
 Genotype 2a    AYDVDMVDANLF----MGGDV---TRIESESKVVVLDSLDPMAEER-SDLEPSIPSE
                AYDVDMVDANLF----MGGDV---TRIESESKVVVLDSLDPMAEEK-SDLEPSIPSE
                TYDVDMVDANLF----MGGDV---TRIESESKVVVLDSLDPMAEER-SDLEPSIPSE
                TYDVDMVDANLF----MGGDV---TRIESESKVVVLDSLDPMAEER-SDLEPSIPSE
                TYDVDMVDANLF----MGGDV---TRIESESKVVVLDSLDPMAEDR-SDHEPSIPSE
                TYDVDMVDASLF----MRGDV---TRIESESKVVVLDYLDPMVEET-SDREPSIPSE
                TYDMDMVEANLF----MGGDV---TRIESESKVVVLDSLDSMVEER-SDLEPSIPSE
                TYDVDMVDANLF----MGGGV---IRIESESKVVVLDSLDSMTEEE-GDLEPSVPSE
                AHDVDMVDANLF----MGGDV---TRIESESKVVVLDSLDPMAEER-SDHEPSIPSE
                AYDVDMVDANLF----MRGDV---TRIESESKVVFLDYLDPVVEEE-RDLEPSIPSE
                AYDVDMVDANLF----MRGEV---TRIESESKVVILDYLDPMTAEE-SDLEPSIPSE
                AHDVDMVDANLF----MGGGE---VWIESESKVVVLDSLDSMAEEK-SDLEPSIPSE

                SPDVDLIEANLLWQQVMGGNI---TRVESENKVVILDSFVPLRAEEDEREISTPAEI
Genotype 1b     SPDADLIEANLLWQQEMGGNI---TRVESENKVVILDSFEPLRAEEGEREVSVPAEI
                PPDIDLIEANLLWRQEMGGNI---TRVESENKVVILDSFDPLRTEEDEGEISVAAEI
                SPDVDLIEANLLWRQEMGGNI---TRVESENKVVVLDSFEPLRAEGDENEISIAAEI
                SPDADLIEANLLWRQEMGGNI---TRVESENKVVILDSFDPLRAEEDEREVSVAAEI
                SPDTDLVEANLMWRQVMGGSI---TRVESENKVVILDSFVPLRTEEDEREVSVPAEI
                SPDVDLLEANLLWQQEMGGNI---TRVESEKKVVILDSFEPLRAEEDEREMSVPAEI
                SPDADLIEANLLWRQEMGGNI---TRVESENKVVILDSFDPIRAVEDEREISVPAEI
    PKR         RFGMDFKEIELIGSGGFGQVFKAKHRIDGKTYVIKRVKYNNE---KAEREVKALAKL
```

(2a)

ISDR

```
D902081b        PSLKATCTTHHDSPDADLIEANLLWRQEMGGNITRVESENKVVILDSFDPIRAVEDEREISVPAEI
AB1913331b      PSLKATCTTHHDSPDADLIEANLLWRQEMGGNITRVESENKVVILDSFDPLRAEEDEREVSVPAEI
AF4832691b      PSLKATCTTHHDSPDADLIEANLLWRQEMGGNITRVESENKVVILDSFDPLRAEEDEREVSVPAEI
EU1553311b      PSLKATCTTRHDSPDADLIEANLLWRQEMGGNITRVESENKVVILDSFDPLRAEEDEREISVPAEI
EF4074981b      PSLKATCTTHHDSPDADLIEANLLWRQEMGGNITRVESENKVVILDSFDPLRAEEDDREVSVPAEI
U454761b        PSLKATCTTRHDSPDADLIEAHLLWRQEMGGNITRVESENKVVILDSFDPLRAEEDDREVSVPAEI
D144841b        PSLKATCTIHHDSPDADLIEANLLWRQEMGGNITRVESENKVVILDSFEPIRAEEDEREVSVPAEI
AF2077621b      PSLKATCTTHRDSPDVDLIEANLLWRQEMGGNITRVESENKVVILDSFEPLRAEEDEREPSVPAEI
AF034151        PSLKATCTTRHDSPDADLIEANLLWRQEMGGNITRVESENKVVILDSFDPLHAEEDEKEVSVPAEI

HCV_3a_NZL1     PSLKATCQTHRPHPDAELVDANLLWRQEMGSNITRVESETKVVVLDSFEPLRAETDDVEPSVAAEC
NR10_3a         PSLKATCQTHRPHPDAELVDANLLWRQEMGSNIVRVESETKVVVLDSFEPLRAETDDAELSVAAEC
NR2_3a          PSLKATCQTHRPHPDAELVDANLLWRQEMGSNITRVESETKVVILDSFEPLRAETDDAELSVAAEC
NR4_3a          PSLKATCQTHRPHPDAELVDANLLWRQEMGSNITRVESETKVVILDSFEPLRAETDDDELSVAAEC
NR6_3a          PSLKATCQTHRPHPDAELVDANLLWRQEMGSNITRVESETKVVILDSFEPLKAENDDVELSVAAEC
NR3_3a          PSLKATCQTHRPHPDAELVDANLLWRQEMGSNITRVESETKVVILDSFEPLRAETDDVELSVAAEC
DQ4308203a      PSLKATCQTHRPHPDVELVDANLLWRQEMGSNITRVESETKVVILDSFEPLRAEPDDIELSVAAEC
AY9564673a      PSLKATCQTHRPHPDAELVDANLLWRQEMGSNITRVESETKVVILDSFEPLRAEPDDTELSVAAEC
GQ3562023a      PSLKATCQTHRPHPDAELVDANLLWRQEMGSNITRVESETKVVILDSFEPLKAETDDTELSVAAEC
GQ3562013a      PSLKATCQTHRPHPDAELVDANLLWRQEMGSNITRVESETKVVILDSFEPLRAETDDAELSVAAEC
DQ4375093a      LSLKATCQTHSPYPDAELVDANLLWRQEMGSNITRVESETKVVILDSFEPLRAETDDAELSVAAEC
GQ3562123a      PSLKATCQTHRPHPDAELVDANLLWRQEMGSYITRLESETEVVVLDSFEPLRAETDDTELSVTAEC
GQ3562133a      PSLKATCQTHRPHPDAELVDANLLWRQEMGSNITRVESETKVVILDSFEPLRAETDDNELSVAAEC
```

V3 REGION

```
D902081b        GS-------SGSSAVDSGTATGPPDQASDDGDKG
AB1913331b      GS-------SGSSAVDSGTATAPPDGPSDDGDAG
AF4832691b      GS-------SGSSAVDSGTATAPPDQSPDDGDAG
EU1553311b      GS-------SESSAVDSGTATAPPDQPSDNGDTG
EF4074981b      GS-------SESSAVDSGTATIPPDQALDDGDKG
U454761b        GS-------SESSAVDSGTATAPPGQSSDDVDTG
D144841b        GG-------SGSSAADSGTATAPPDQTSDDGDKE
AF2077621b      GS-------SGSSAVDSGTATAPPDQASNDGDKE
AF034151        GS-------SGSSAVDSGTASAPPDQSTDNGDTG

HCV_3a_NZL1     PSSKPQEENSSSSGVDTQSSTTSKVPPSPGGE--
NR10_3a         SSSEPQEENSSSSGVDTQSSTTSKVPPSPGGE--
NR2_3a          PSSKPQEENSSSSGVDTQSSTTSKVPPSPGGE--
NR4_3a          PSSKPQEENSSSSGVDTQSSTTSKVPPSPGGE--
NR6_3a          PSPKPQEENSSSSGVDTQSSTTSRVPPSPAGE--
NR3_3a          PSPKPQEENSSSSGVDTQSSTTSRVPPSPAGE--
DQ4308203a      PSSEPQGENSSSSGVDTQSSTASKVPPSSGGE--
AY9564673a      SSSKPQEEDSSSSGVDTQSSTTSKVPPPPEEE--
GQ3562023a      PSAKSQEESSSSSGVGTQSSTAPKVPPSSEEE--
GQ3562013a      PPPKPQEENSSSSGVDTQSSTTSKAPPSPREE--
DQ4375093a      PSSKLQEENSSSSGVDTQPSTTSNVSPSPGGE--
GQ3562123a      PSSNLQEKDSSSSGVDAQSSTTSKVPPSSRGE--
GQ3562133a      PSSKPQEETSSSSGVDTQSSTTSKAPPSPEGE--
```

(2b)

ISDR

```
D90208          PSLKATCTTHHDSPDADLIEANLLWRQEMGGNITRVESENKVVILDSFDPIRAVEDEREISVPAEI
AB1913331b      PSLKATCTTHHDSPDADLIEANLLWRQEMGGNITRVESENKVVILDSFDPLRAEEDEREVSVPAEI
AF4832691b      PSLKATCTTHHDSPDADLIEANLLWRQEMGGNITRVESENKVVILDSFDPLRAEEDEREVSVPAEI
EU1553311b      PSLKATCTTRHDSPDADLIEANLLWRQEMGGNITRVESENKVVILDSFDPLRAEEDEREISVPAEI
EF4074981b      PSLKATCTTHHDSPDADLIEANLLWRQEMGGNITRVESENKVVILDSFDPLRAEEDDREVSVPAEI
U454761b        PSLKATCTTRHDSPDADLIEAHLLWRQEMGGNITRVESENKVVILDSFDPLRAEEDEREVSVPAEI
D144841b        PSLKATCTIHHDSPDADLIEANLLWRQEMGGNITRVESENKVVILDSFEPIRAEEDEREVSVPAEI
AF2077621b      PSLKATCTTHRDSPDVDLIEANLLWRQEMGGNITRVESENKVVILDSFEPLRAEEDEREPSVPAEI
AF034151        PSLKATCTTRHDSPDADLIEANLLWRQEMGGNITRVESENKVVILDSFDPLHAEEDEKEVSVPAEI

AB0476392a      PSLRATCTTHSNTYDVDMVDANLL----MEGGVAQTEPESRVPVLDFLEPMAEEESDLEPSIPSEC
D009442a        PSLRATCTTHGKAYDVDMVDANLF----MGGDVTRIESESKVVVLDSLDPMVEERSDLEPSIPSEY
HCV_2a          PSLRATCTTHGKAYDVDMVDANLF----MGGDVTRIESESKVVVLDSLDPMVEERSDLEPSIPSEY
AB0476412a      PSLRATCTTHGKTHDVDMVDANLF----MGGDVTRIESESKVVVLDSLDPMVEEEGDLEPSIPSEY
AF1690022a      PSLRATCATHGKTYDVDMVDANLF----MGGDVTRIESESKVVVLDSLDPMAEERSDLEPSIPSEY
AF2384852a      PSLRATCTTHGKTYDVDMVDANLF----MGGDVTRIESESKVVVLDSLDPMAEERSDLEPSIPSEY
AB0476432a      VSLRATCATHGEAYDVDMVDANLF----MRGEVTRIESESKVVILDYLDPMTAEESDLEPSIPSEY
AY7464602a      PSLRATCTTYGRAHDVDMVDANLF----MGGGEVWIESESKVVVLDSLDSMAEEKSDLEPSIPSEY
```

V3 REGION

```
D90208            G----------SSGSSAVDSGTATGPPDQASDDGDKG
AB1913331b        G----------SSGSSAVDSGTATAPPDGPSDDGDAG
AF4832691b        G----------SSGSSAVDSGTATAPPDQSPDDGDAG
EU1553311b        G----------SSESSAVDSGTATAPPDQPSDNGDTG
EF4074981b        G----------SSESSAVDSGTATIPPDQALDDGDKG
U454761b          G----------SSESSAVDSGTATAPPGQSSDDVDTG
D144841b          G----------GSGSSAADSGTATAPPDQTSDDGDKE
AF2077621b        G----------SSGSSAVDSGTATAPPDQASNDGDKE
AF034151          G----------SSGSSAVDSGTASAPPDQSTDNGDTG

AB0476392a        GQPPSSGDAGSSTGAGAAESGGPT-SPGEP-----AP
D009442a          GQPPPSGDSGLSTGADAADSGSRT-PPDEL-----AL
HCV_2a            GQPPPSGDSGLSTGADAADSGSRT-PPDEL-----AL
AB0476412a        GQPPPSGDSGLSTGADAADSGSRT-PPDES-----AL
AF1690022a        GQPPPSGDSGLSTGADAADSGGQA-SPDEL-----AL
AF2384852a        GQPPPSGDSGLSTGADAADSGGRT-PPDEL-----AL
AB0476432a        GQAPPSGESGLSTGADAADSGGRT-PPDES-----AL
AY7464602a        GQPPPSGNSGLSTGADAAGSGGRT-PPDEL-----AL
```

(2c) ISDR

```
D90208          PSLKATCTTHHDSPDADLIEANLLWRQEMGGNITRVESENKVVILDSFDPIRAVEDEREISVPAEI
AB1913331b      PSLKATCTTHHDSPDADLIEANLLWRQEMGGNITRVESENKVVILDSFDPLRAEEDEREVSVPAEI
AF4832691b      PSLKATCTTHHDSPDADLIEANLLWRQEMGGNITRVESENKVVILDSFDPLRAEEDEREVSVPAEI
EU1553311b      PSLKATCTTRHDSPDADLIEANLLWRQEMGGNITRVESENKVVILDSFDPLRAEEDEREISVPAEI
EF4074981b      PSLKATCTTHHDSPDADLIEANLLWRQEMGGNITRVESENKVVILDSFDPLRAEEDDREVSVPAEI
U454761b        PSLKATCTTRHDSPDADLIEAHLLWRQEMGGNITRVESENKVVILDSFDPLRAEEDEREVSVPAEI
D144841b        PSLKATCTIHHDSPDADLIEANLLWRQEMGGNITRVESENKVVILDSFEPIRAEEDEREVSVPAEI
AF2077621b      PSLKATCTTHRDSPDVDLIEANLLWRQEMGGNITRVESENKVVILDSFEPLRAEEDEREPSVPAEI
AF034151        PSLKATCTTRHDSPDADLIEANLLWRQEMGGNITRVESENKVVILDSFDPLHAEEDEKEVSVPAEI

GQ3562133a      PSLKATCQTHRPHPDAELVDANLLWRQEMGSNITRVESETKVVILDSFEPLRAETDDNELSVAAEC
HCV_3a_NZL1     PSLKATCQTHRPHPDAELVDANLLWRQEMGSNITRVESETKVVVLDSFEPLRAETDDVEPSVAAEC
NR10_3a         PSLKATCQTHRPHPDAELVDANLLWRQEMGSNIVRVESETKVVVLDSFEPLRAETDDAELSVAAEC
NR2_3a          PSLKATCQTHRPHPDAELVDANLLWRQEMGSNITRVESETKVVILDSFEPLRAETDDAELSVAAEC
NR4_3a          PSLKATCQTHRPHPDAELVDANLLWRQEMGSNITRVESETKVVILDSFEPLRAETDDDELSVAAEC
NR6_3a          PSLKATCQTHRPHPDAELVDANLLWRQEMGSNITRVESETKVVILDSFEPLKAENDDVELSVAAEC
NR3_3a          PSLKATCQTHRPHPDAELVDANLLWRQEMGSNITRVESETKVVILDSFEPLRAETDDVELSVAAEC
DQ4308203a      PSLKATCQTHRPHPDVELVDANLLWRQEMGSNITRVESETKVVILDSFEPLRAEPDDIELSVAAEC
AY9564673a      PSLKATCQTHRPHPDAELVDANLLWRQEMGSNITRVESETKVVILDSFEPLRAEPDDTELSVAAEC
GQ3562023a      PSLKATCQTHRPHPDAELVDANLLWRQEMGSNITRVESETKVVILDSFEPLKAETDDTELSVAAEC
GQ3562013a      PSLKATCQTHRPHPDAELVDANLLWRQEMGSNITRVESETKVVILDSFEPLRAETDDAELSVAAEC
DQ4375093a      LSLKATCQTHSPYPDAELVDANLLWRQEMGSNITRVESETKVVILDSFEPLRAETDDAELSVAAEC
GQ3562123a      PSLKATCQTHRPHPDAELVDANLLWRQEMGSYITRLESETEVVVLDSFEPLRAETDDTELSVTAEC
AB0476392a      PSLRATCTTHSNTYDVDMVDANLL----MEGGVAQTEPESRVPVLDFLEPMAEEESDLEPSIPSEC
D009442a        PSLRATCTTHGKAYDVDMVDANLF----MGGDVTRIESESKVVVLDSLDPMVEERSDLEPSIPSEY
HCV_2A          PSLRATCTTHGKAYDVDMVDANLF----MGGDVTRIESESKVVVLDSLDPMVEERSDLEPSIPSEY
AB0476412a      PSLRATCTTHGKTHDVDMVDANLF----MGGDVTRIESESKVVVLDSLDPMVEEEGDLEPSIPSEY
AF1690022a      PSLRATCATHGKTYDVDMVDANLF----MGGDVTRIESESKVVVLDSLDPMAEERSDLEPSIPSEY
AF2384852a      PSLRATCTTHGKTYDVDMVDANLF----MGGDVTRIESESKVVVLDSLDPMAEERSDLEPSIPSEY
AB0476432a      VSLRATCATHGEAYDVDMVDANLF----MRGEVTRIESESKVVILDYLDPMTAEESDLEPSIPSEY
AY7464602a      PSLRATCTTYGRAHDVDMVDANLF----MGGGEVWIESESKVVVLDSLDSMAEEKSDLEPSIPSEY
```

V3 REGION

```
D90208          GS-------SGSSAVDSGTATG---PPDQASDDGDKG
AB1913331b      GS-------SGSSAVDSGTATA---PPDGPSDDGDAG
AF4832691b      GS-------SGSSAVDSGTATA---PPDQSPDDGDAG
EU1553311b      GS-------SESSAVDSGTATA---PPDQPSDNGDTG
EF4074981b      GS-------SESSAVDSGTATI---PPDQALDDGDKG
U454761b        GS-------SESSAVDSGTATA---PPGQSSDDVDTG
D144841b        GG-------SGSSAADSGTATA---PPDQTSDDGDKE
AF2077621b      GS-------SGSSAVDSGTATA---PPDQASNDGDKE
AF034151        GS-------SGSSAVDSGTASA---PPDQSTDNGDTG

GQ3562133a      PSSKPQEETSSSSGVDTQSSTTSKAPPSPE-----GE
Hcv_3a_NZL1     PSSKPQEENSSSSGVDTQSSTTSKVPPSPG-----GE
NR10_3a         SSSEPQEENSSSSGVDTQSSTTSKVPPSPG-----GE
NR2_3a          PSSKPQEENSSSSGVDTQSSTTSKVPPSPG-----GE
NR4_3a          PSSKPQEENSSSSGVDTQSSTTSKVPPSPG-----GE
NR6_3a          PSPKPQEENSSSSGVDTQSSTTSRVPPSPA-----GE
NR3_3a          PSPKPQEENSSSSGVDTQSSTTSRVPPSPA-----GE
DQ4308203a      PSSEPQGENSSSSGVDTQSSTASKVPPSSG-----GE
AY9564673a      SSSKPQEEDSSSSGVDTQSSTTSKVPPPPE-----EE
GQ3562023a      PSAKSQEESSSSSGVGTQSSTAPKVPPSSE-----EE
GQ3562013a      PPPKPQEENSSSSGVDTQSSTTSKAPPSPR-----EE
DQ4375093a      PSSKLQEENSSSSGVDTQPSTTSNVSPSPG-----GE
GQ3562123a      PSSNLQEKDSSSSGVDAQSSTTSKVPPSSR-----GE
AB0476392a      GQPPSSGDAGSSTGAGAAES-GGPTSPGEP-----AP
D009442a        GQPPPSGDSGLSTGADAADS-GSRTPPDEL-----AL
HCV_2a          GQPPPSGDSGLSTGADAADS-GSRTPPDEL-----AL
AB0476412a      GQPPPSGDSGLSTGADAADS-GSRTPPDES-----AL
AF1690022a      GQPPPSGDSGLSTGADAADS-GGQASPDEL-----AL
AF2384852a      GQPPPSGDSGLSTGADAADS-GGRTPPDEL-----AL
AB0476432a      GQAPPSGESGLSTGADAADS-GGRTPPDES-----AL
AY7464602a      GQPPPSGNSGLSTGADAAGS-GGRTPPDEL-----AL
```
